# SARS-CoV-2 memory response in non-hospitalised cases: immunology in the context of a population-based cohort study

**DOI:** 10.12688/wellcomeopenres.22942.1

**Published:** 2024-10-11

**Authors:** Ruth E. Mitchell, Milla Kibble, Marianna Santopaolo, Emily Milodowski, Holly E. Baum, Ore Francis, Alice Halliday, Elizabeth Oliver, Benjamin Hitchings, Amy C. Thomas, Susan M. Ring, Karen Ho, Kate Northstone, Katrina Entwistle, Begonia Morales-Aza, Jennifer Oliver, Anu Goenka, Andrew Davidson, Adam Finn, Linda Wooldridge, Laura Rivino, Nicholas J. Timpson

**Affiliations:** 1 Population Health Sciences, Bristol Medical School, University of Bristol, Bristol, England, UK; 2 MRC Integrative Epidemiology Unit, University of Bristol, Bristol, England, UK; 3 Department of Applied Mathematics and Theoretical Physics, University of Cambridge, Cambridge, England, UK; 4 Department of Twin Research and Genetic Epidemiology, King's College London, London, England, UK; 5 School of Cellular and Molecular Medicine, University of Bristol Faculty of Medical and Veterinary Sciences, Bristol, England, UK; 6 Faculty of Health and Life Sciences, Bristol Veterinary School, University of Bristol, Langford, England, UK; 7 Bristol Vaccine Centre, University of Bristol, Bristol, England, UK; 8 Department of Paediatric Immunology and Infectious Diseases, Bristol Royal Hospital for Children, Bristol, UK

**Keywords:** SARS-CoV-2, COVID-19, Memory immune response, ALSPAC

## Abstract

**Background:**

The study of non-hospitalised COVID-19 cases provides a context for improved understanding of the immune response to existing and new infections. Population-based cohorts provide a unique opportunity to do this in relation to rich longitudinal pre- and pan-pandemic data. The Avon Longitudinal Study of Parents and Children (ALSPAC) is a prospective population-based cohort study which recruited pregnant women in 1990-1992 and has subsequently followed participants for over 30 years.

**Methods:**

A study comprising three clinic visits was implemented, in response to the COVID-19 pandemic, amongst ALSPAC participants to measure SARS-CoV-2 specific humoral and cellular responses longitudinally. Here we present data from the first clinic in December 2020 before the start of the UK vaccination campaign and examine associations with a set of exemplar pre- and pan-pandemic health factors.

**Results:**

We observed humoral and cellular memory immune responses to SARS-CoV-2 infection in mild cases of COVID-19 up to 9 months post-infection. Symptomatic infection elicited a memory immune response of greater magnitude, though there was variation in response in both asymptomatic and symptomatic individuals. We examined health factors associated with severe COVID-19 and found that cardio-metabolomic, respiratory and immune-related health factors associate with a memory immune response of higher magnitude. For example, in older participants (mean age 58 years), higher BMI was associated with an immune memory response of greater magnitude, particularly with anti-S and anti-N binding antibodies.

**Conclusions:**

We set out to illustrate the use of cohort studies to deliver detailed immunological data and to provide example analyses of how life course health factors can be examined in relation to the immune response following a widespread and novel infection. We expanded this assessment to include longitudinally assessed traits, opening up the potential for the more common use of longitudinal population studies for the better understanding the aetiology of infection outcome.

## Plain Language Summary

Although SARS-CoV-2 infections have resulted in severe disease, hospitalisation and death for millions globally during the COVID-19 pandemic, the majority of infections have been mild or asymptomatic. In this study, we examine the immune responses of people unvaccinated and with mild or asymptomatic SARS-CoV-2 infection and see how these responses are associated with their health prior to SARS-CoV-2 infection. Study participants were all part of the Avon Longitudinal Study of Parents and Children (ALSPAC), with ages ranging from 27 to 70. We found evidence suggesting that cardio-metabolomic, respiratory and immune-related health associates with a memory immune response of higher magnitude. These associations remained when adjusting for symptom status. Whilst only exemplar associations were examined here, relationships suggest that whilst morbidity may be correlated with immune response, life course health factor associations with immunological response potentially exist in addition to this and may be connected to infection outcomes.

## Introduction

Infection with severe acute respiratory syndrome coronavirus 2 (SARS-CoV-2) spread rapidly across the globe following its emergence in December 2019 causing an acute respiratory disease named Coronavirus disease 2019 (COVID-19) ^1^. As this was a novel coronavirus (CoV) ^2^, the initial wave of infection was in immunologically naïve individuals. A spectrum of different clinical scenarios were observed ranging from completely asymptomatic to severely symptomatic, with substantial morbidity and mortality reported globally ^3^. It is now clear that upon primary exposure to SARS-CoV-2, an innate immune response is mounted ^4^ and an adaptive immune response is primed, which leads to the development of immunological memory and offers protection to the host against severe disease on subsequent exposure to SARS-CoV-2 ^5, 6^. Multiple arms of the adaptive immune system contribute to immunological memory and protection from severe COVID-19, including CD4 ^+^ T-cells, CD8 ^+^ T-cells and B-cells (which produce antibodies) ^5, 7– 10^. Therefore, it is important to incorporate assays to assess each of these compartments into any study of the SARS-CoV-2 specific memory response.

Studies have focused on assessing the magnitude of the circulating antibody response due to the feasibility of performing serological assays on large numbers of people ^11^. The majority of individuals infected with SARS-CoV-2 have been shown to seroconvert and develop a neutralising antibody response. Although some waning of this response has been reported, circulating antibodies remain detectable after 8 months post infection ^11^. Studies have also reported memory T-cell responses in individuals following SARS-CoV-2 infection in the absence of detectable antibodies ^8, 12– 14^. In addition, it has been suggested that pre-existing cross-reactive T-cell responses may play a role in protection against symptomatic or severe disease in highly exposed individuals ^13^. This suggests that T-cells may play an important role in protective immunity ^15, 16^.

Previous focus on hospitalised patients is clearly a lens onto those who have experienced severe disease. Whilst useful, the study of non-hospitalised COVID-19 cases is also important for understanding the memory immune response in the majority of individuals who will have been infected with SARS-CoV-2. Furthermore, although studies into factors that might influence the magnitude and nature of the memory immune response have been undertaken, they have focused on factors present at the time of infection ^17, 18^ and not health factors in childhood or in the years preceding infection. The utility of longitudinal studies thus comes to the forefront in the context of infectious disease aetiology and response/outcome monitoring.

The Avon Longitudinal Study of Parents and Children (ALSPAC) provides an extensive resource to investigate immunological memory to SARS-CoV-2 in relation to health, given the availability of rich longitudinal pre-pandemic data. ALSPAC is a unique multi-generational study comprising ‘G0’: the cohort of original pregnant women recruited to the study between 1991–1992, with the biological fathers and other carers/partners; ‘G1’: the cohort of index children; and ‘G2’: the cohort of offspring of the index children. The study biological, genetic and phenotypic data across all three of these generations spanning over 30 years ^19– 22^. Throughout the COVID-19 pandemic, ALSPAC captured information across G0 and G1 generations providing a snapshot of infection in a higher risk (the G0 cohort; mean age ~59 years) and lower risk (the G1 cohort; mean age ~28 years) non-hospitalised general population ^23– 25^. This provides a unique opportunity to investigate the influence of prior health factors on the immune memory response to natural infection with SARS-CoV-2 and how these responses relate to symptoms in a non-hospitalised general population.

We aimed to investigate, prior to the rollout of COVID-19 vaccines in the UK, the magnitude and nature of the memory immune response following mild cases of natural SARS-CoV-2 infection. To undertake this work, ALSPAC was rapidly mobilised and 325 participants were invited to participate in a clinic visit in December 2020, during which blood samples were taken and questionnaires completed. This first clinic visit forms part of a broader study comprising three sequential visits (December 2020, March 2021, June 2021), designed to describe the magnitude and durability of immunological memory to SARS-CoV-2 infection and to better understand factors that might be important in the aetiology of COVID-19 and other infections. We also aimed to demonstrate the potential utility of longitudinal population studies in assessing how the immune response to a new pathogen varies according to the presence or absence of symptoms at the time of infection and whether the immune response is associated with prior health factors.

## Methods

### ALSPAC longitudinal population cohort

Details of the ALSPAC study (also known as Children of the 90s by participants and the public) have been well documented ^19– 22^. Briefly, pregnant women resident in Avon, UK with expected dates of delivery between 1st April 1991 and 31st December 1992 were invited to take part in the study. The initial number of pregnancies enrolled was 14,541 with 13,988 children who were alive at one year of age. The initial cohort was later expanded when children were approximately age 7, to give a total of 15,447 pregnancies, with 14,901 children alive at one year (generation G1). This provides a total of 14,833 unique women (G0 mothers) enrolled in ALSPAC as of September 2021. 12,113 G0 partners have been in contact with the study by providing data and/or formally enrolling when this started in 2010. 3,807 G0 partners are currently enrolled. The women, the children arising from their pregnancies, and their partners have been followed up intensively over three decades, providing detailed historical data and biological samples for these participants with consent for use in future research. The study website contains details of all the data that is available through a fully searchable data dictionary and variable search tool ( http://www.bristol.ac.uk/alspac/researchers/our-data/).

### Study design and participant description

**Figure 1.**
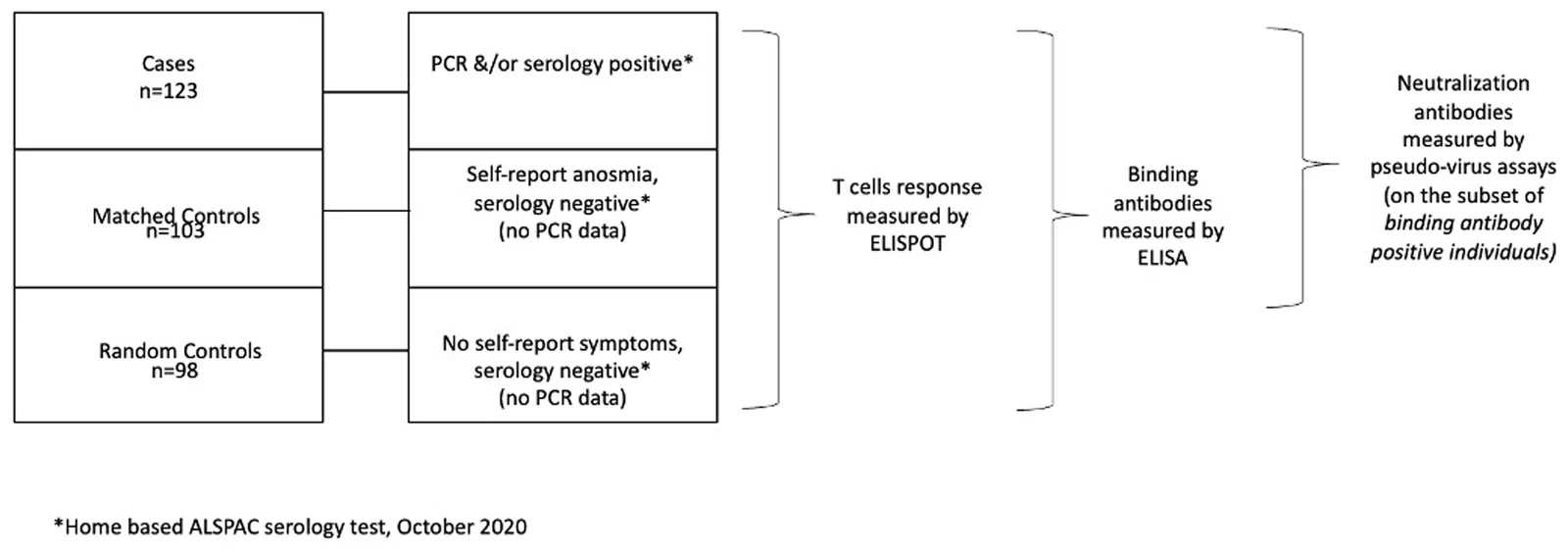
Schematic of study design. Individuals from the Avon Longitudinal Study of Parents and Children (ALSPAC) were selected based on the results of serological testing in October 2020. SARS-CoV-2-specific T-cell response measured as IFN-γ secreted in response to stimulation of PBMCs with Spike (pool-1 and 2) N, M, non-structural proteins (NSP1+2, NSP3A, NSP3B, NSP3C, NSP4, NSP5+6, NSP7-11, NSP12A, NSP12B, NSP13, NSP14, NSP15-16), accessory peptide pools (ORF3, ORF6, ORF7, ORF8) and Envelope protein (E) and SARS-CoV-2-specific antibody response to anti-Spike and anti-Nucleocapsid Pan Ig antibody were measured on all cases and controls. Neutralisation of Pseudotyped VSV by serum was performed on the subset of binding Pan Ig antibody positive individuals.

Recruitment to this study was undertaken within ALSPAC participants and undertaken at a time when limited information was available as to the relationship between the immune response and symptom profile. In October 2020, ALSPAC undertook 4819 serological SARS-CoV-2 lateral flow tests (LFTs), where presence of antibodies was considered a positive test, on G0 and G1 cohort participants ^25^ as well as administering regular COVID-19 online questionnaires to participants during the pandemic ^23– 27^. At the time, cumulative confirmed infection levels across the UK were approximately 1% ^28^. Based on the results of this serology testing and/or positive PCR test results from data linked from UK Health Security Agency (UKHSA) formerly known as Public Health England (PHE), 124 cases of previous SARS-CoV-2 infection were recruited for an initial face to face interaction (data and sample collection) in November and December 2020. Serology tests were also used to recruit two control groups: 103 control individuals without evidence of previous SARS-CoV-2 infection (i.e. negative serological LFT and no previously documented positive PCR test) who were age, sex and symptom (anosmia) matched to cases (“matched controls”) to encompass individuals with similar symptoms as stability of antibodies against SARS-CoV-2 measured by LFT was unknown at the time; and 98 controls without any evidence of previous SARS-CoV-2 infection or any documented anosmia (“random controls”) ( Figure 1). Priority was given to participants with pre-pandemic measures of BMI and lung function (objectively assessed at the age of 24 years in G1) and previous biological samples. Individuals taking anti-coagulants (e.g. warfarin) or with a known clotting disorder were excluded from the study.

### Questionnaire, clinic and immunological measurements

At the first clinic in November/December 2020 and each subsequent clinic visit, a questionnaire was completed by participants. Clinical measures taken on site comprised of blood samples (4x10ml EDTA, 1x5ml Serum); saliva sample, physical measures (height, weight); respiratory function testing; and a urine sample. A detailed description of the questionnaire and clinical measures can be found in the Supplementary document and Supplementary table 1, which can be found as *Extended data* ^29^. Further, the supplementary document details the protocols for the analysis of SARS-CoV-2 antibodies and T-cell responses which include IFN-γ enzyme-linked immunospot (ELISpot) assay, Enzyme-linked immune absorbent spot assay (ELISA), and Pseudo-virus Neutralisation assays.

### Definition of analysis cases

For the in-depth analysis of immune memory to SARS-CoV-2, which could only be carried out for participants who had previously contracted COVID-19, we wanted to maximise the case sample. Thus, in addition to the 114 participants in the case group (which included individuals found to be seropositive and seronegative at the clinic visit), we added all participants from the matched or random control groups who had positive binding antibodies detected at the clinic by serum ELISA testing (against S or N, or both). This generated an augmented case group (n=139) which we refer to hereon as ‘analysis cases’.

### Correlation between the immune memory variables

The immune variables were inverse normal rank transformed to ensure a normal distribution using all analysis cases. The correlation coefficient (r ^2^) between immune variables was calculated using multivariate ordinary least squares linear regression, with covariates age and sex.

### Associations between symptoms and immune memory variables

Analyses were performed within generations and immune variables were inverse normal rank transformed to ensure a normal distribution using all analysis cases from within the appropriate generation, G0 or G1. Three different measures of symptom severity were used in this study. Each derived using responses from COVID-19 questionnaires that were rolled out during the COVID-19 pandemic, including questions on presence and absence of individual symptoms by month. A detailed description of each method, as well as a comparison between the methods, can be found in the Supplementary document. In brief, the first measure assigns an analysis case to have been either asymptomatic or symptomatic based on an algorithm (the Menni algorithm ^30^), which calculates occurrence of COVID-19 infection based on four symptoms (loss of smell and taste; severe or significant persistent cough; severe fatigue and skipped meals; age and sex) and predicts probable infection on a monthly basis up to (but not including) December 2020 ^23^. Secondly, an analysis case was predicted to have lost their taste or smell if the participant reported loss of taste or smell in the period March-December 2020. Thirdly, for most analysis cases (112/139), we did not know the date of COVID-19 infection. Therefore, we could not simply assign the number of symptoms by checking in the month of infection. Instead, for each individual and each month from March 2020 (inclusive) onwards, we counted the number of symptoms reported in each month out of a list of 12–13 standard COVID-19 symptoms ^30, 31^. We call the month where the first instance of a maximum jump in number of symptoms occurs as the “month of infection by symptoms” and take the number of symptoms associated with a COVID-19 infection to be the number of symptoms recorded in the month of infection by symptoms.

Comparisons of immune variable levels for asymptomatic versus symptomatic cases and loss versus no loss of taste or smell were carried out using a Student’s t test. Correlation between number of symptoms and immune variables was calculated using multivariate ordinary least squares linear regression, with covariates age and sex.

### Analysis of impact of prior health measures on the SARS-CoV-2 memory response

For each of the antigen-specific immune responses measured, we chose to look for associations with a number of prior health variables selected given their links to risk factors for acute disease. For the G0 generation, health variable names are appended with the year in which the data was collected. For the younger G1 generation, variable names are appended with the approximate age at which the data was collected (since the ages of the G1 cohort are all roughly the same). The precise pre and pan-pandemic ALSPAC datasets used are listed in the Supplementary document.

We ran linear regression models of each immune response variable against each prior health variable. For the discrete variables, we took no disease as the reference. Covariates in the initial models were age and sex, with additional analyses on the effect of symptoms including a third covariate (asymptomatic/symptomatic as defined using the Menni algorithm, number of reported symptoms, or loss of taste or smell). The associations discussed below persist after the addition of symptoms as a covariate and can be found for the extended set of immune variables in the Supplementary file. Health variable counts for each generation are presented in Supplementary table 4.

For the BMI trajectories, each individual was assigned into either a high, intermediate or low group of each immune variable. Natural cubic spline linear mixed-effects models were fitted using the ‘lme4’ package with three knots placed at quantiles of the age distribution.

Analyses were performed using R version 4.2.1 (R Core Team 2021; https://www.R-project.org/) and GraphPad Prism ( https://www.graphpad.com/).

## Results

### Participant characteristics

Individuals across the cases, matched controls and random controls were skewed towards the younger ages, with more female participants across both generations, and were predominantly in good general health, with vigorous exercise slightly skewed towards “not limited”, low mood weighted towards “no” or “a little” and tiredness weighted towards “yes” and “a little” ( Table 1; extended variable list in Supplementary Table 1). Information on ethnicity was available for study participants who had also completed the fourth ALSPAC COVID-19 questionnaire, with fewer than five participants being of non-white ethnicity in each group.

**Table 1.** Participant characteristics. *This may include zero. The vast majority of random controls were females.

		Cases N=123 (G1=73; G0=50)	Symptom-matched controls N=103 (G1=51; G0=52)	Random controls N=98 (G1=60; G0=38)
Sex (F/M)	G1 G0	58/15 35/15	37/14 35/17	>50/<5 * >30/<5 *
Age	G1 G0	28.3 (27-29) 58.5 (50-70)	28.3 (27-29) 58.9 (49-70)	28.2 (27-29) 60.3 (53-70)
BMI (mean, range)	G1 G0 Total	26.2 (16.8-49.3) 28.9 (18.1-42.9) 27.2 (16.8-49.3)	25.8 (16.8-50.9) 27.8 (19.5-43.5) 26.8 (16.8-50.9)	25.5 (18.2-37.7) 25.6 (20.7-31.4) 25.5 (18.2-37.7)
Chair rises	(mean, range)	27.2 (13-83)	24.7 (12-57)	26.7 (16-98)
	Lowest oxygen saturation	95.9 (83-100)	96.2 (89-100)	96 (86-100)
	Highest heart rate	99.6 (38-150)	97.1 (53-151)	103.8 (52-154)
General health	Good Poor	117 5	98 5	>90 <5 *
Vigorous exercise	Limited Not limited	50 70	43 60	37 61
Mental health (low mood)	Yes A little No	10 68 43	10 60 33	13 57 28
Tiredness	Yes A little or None	42 80	28 75	28 70

### The SARS-CoV-2 memory response to natural infection

**Figure 2.**
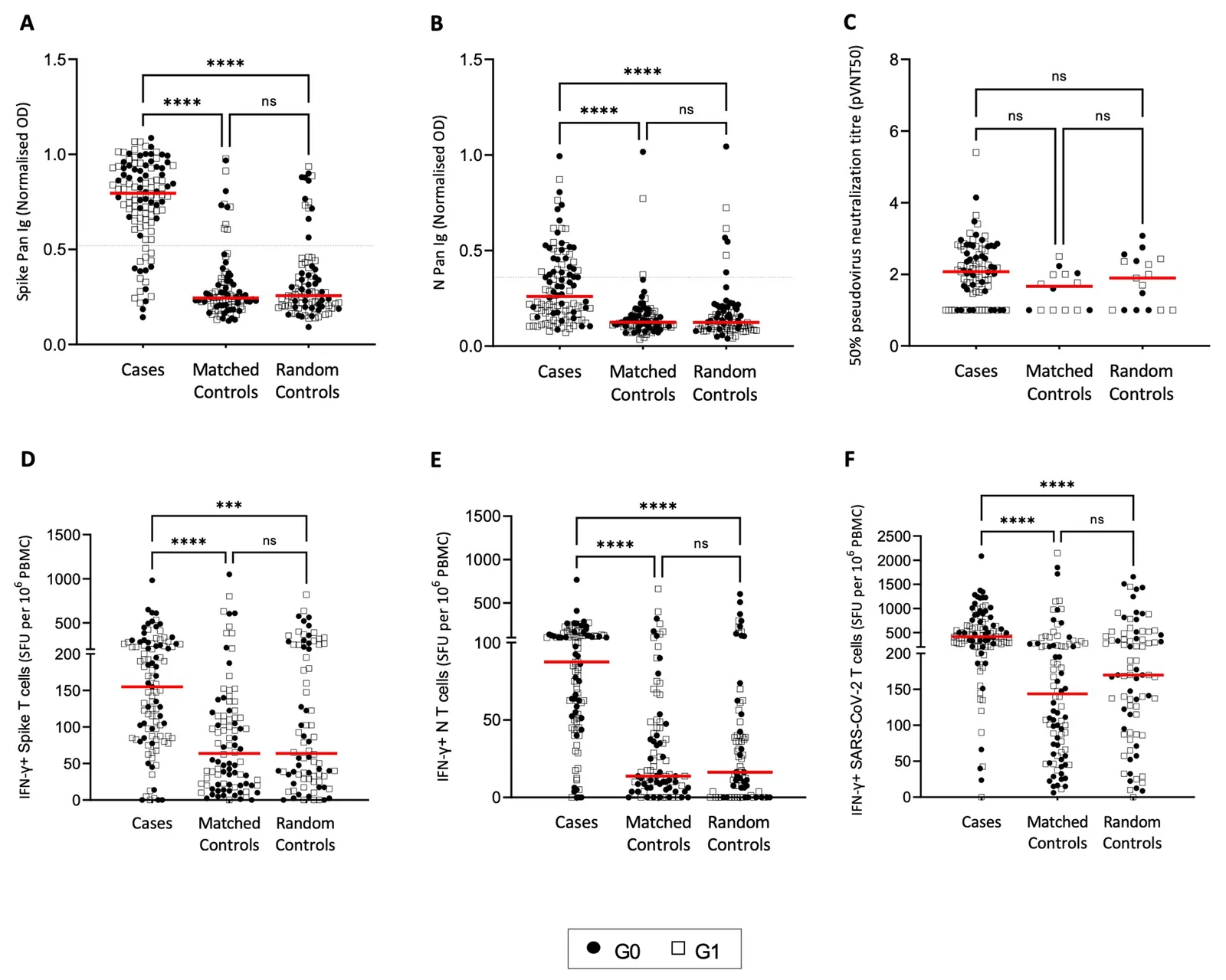
SARS-CoV-2 specific immune response. SARS-CoV-2-specific antibody and T-cell memory responses, stratified by generation; G0 (solid black circles) and G1 (open squares). ( **A**) Serum anti-Spike and ( **B**) anti-Nucleocapsid Pan Ig antibody titres measured by ELISA for cases (n=114), matched controls (n=96) and random controls (n=91). Reference optical density (OD) measurements at 620 nm were subtracted from signal OD at 492 nm, background corrected, and normalised to an internal standard pool. Dotted lines indicate the threshold for each assay, set to achieve 99% specificity. ( **C**) Neutralisation of Pseudotyped VSV by serum. Pseudotype neutralisation assays performed on serum samples, presented as Log 50% neutralisation dilution factors. Non-neutralising samples baselined at 1. Cases (n=96), matched controls (n=14) and random controls (n=17). ( **D**) SARS-CoV-2-specific T-cell response against Spike (pool-1 and 2) and ( **E**) Nucleocapsid (N) peptide pools. ( **F**) Total SARS-CoV-2-specific T-cell responses assessed as the summed response to peptide pools spanning Spike, M, N proteins and non-structural proteins (NSP3b, NSP12A, NSP12B, NSP13 and NSP15-16). T-cell responses in D-F are measured using IFN-γ ELISpot assay after stimulation of PBMCs with overlapping peptides spanning the indicated proteins. Responses are shown for G0 and G1 generations, in cases (n=109), matched controls (n=92) and random controls (n=83). Results are expressed as Spot Forming Units (SFU) relative to 1x10 ^6^ PBMCs after subtraction of values from the unstimulated wells. Negative values after background subtraction were adjusted to zero. Data points represent means of technical duplicates for ELISAs, triplicates for pseudoviral neutralisation, and single observations for ELISpots. Red bars represent the median response within each group. P values were determined using a Kruskal-Wallis test with Dunn’s correction for multiple comparisons (ns P>0.05, * P ≤ 0.05, ** P < 0.01, *** P < 0.001, **** P < 0.0001).

To assess the magnitude of the immunological memory response to SARS-CoV-2, we measured levels of antibodies and magnitude of T-cell responses targeting SARS-CoV-2 in blood samples collected from all participants. Levels of binding antibodies specific for the Spike (S) and Nucleocapsid (N) proteins of SARS-CoV-2 were measured in sera from all participants ( Figures 2A and 2B, Supplementary Figures 1A and 1B). Levels of neutralizing antibodies were subsequently measured on the subset of binding antibody positive individuals ( Figure 2C, Supplementary Figure 1C), using the same samples. The magnitude of T-cell responses (measured as IFN-γ secreted in response to stimulation with pools of overlapping peptides spanning multiple proteins from within the SARS-CoV-2 proteome) were measured on all participants ( Figures 2D–F, Supplementary Figures 2–4). 

### SARS-CoV-2 specific binding and neutralizing antibody responses

Serum samples were analysed using both S and N protein ELISAs, with optical density (OD) values normalised to a standard control. Thresholds for antibody positivity were set and validated using pre-pandemic samples to achieve 99% specificity ^32^. In the case group, most participants (96/114) were shown to be seropositive ( Figures 2A and 2B). Antibodies directed to the S protein were observed more frequently than to the N protein (95/114 and 41/114 respectively) and N responders were almost exclusively also S responders. Antibody levels were comparable between the G0 (average age of 58) and G1 (average age of 28) cohorts and no substantial differences were observed by sex (Supplementary Figures 1A and 1B). Seropositivity rates were lower in the matched and random control groups compared to the case group. Despite the lack of a previous positive PCR test or a previous positive serology LFT at the point of enrolment into the study, a proportion of the participants in the matched and random control groups (25/187) had specific anti-S or anti-N antibodies. This difference is likely suggestive of SARS-CoV-2 infections in the period between the initial screening stage in October and the clinic 1 samples being taken in December, in combination with the increased sensitivity of the ELISAs. There was no substantive difference in antibody positivity rates between matched and random control groups (12/96 and 13/91 respectively).

All available samples displaying anti-SARS-CoV-2 Spike Pan Ig antibody responses above the threshold (≥ 0.52 normalised OD units) were selected for viral pseudotype neutralisation assays. 11 samples immediately below the threshold were also selected to investigate if neutralisation could be observed above and below the threshold. An additional sample, far below the threshold was chosen as a likely negative control. SARS-CoV-2 specific neutralising antibody responses against Spike protein were observed in most individuals selected for pseudovirus neutralisation assays (94/127). Neutralisation was observed in the majority of samples tested from the case group, including an individual from the 11 below-ELISA threshold samples included in this assay and subsequent analysis (75/96). Additionally, most of the selected samples from the matched (8/14) and random control groups (11/17) neutralised pseudovirus ( Figure 2C). Neutralisation across these three groups occurred at similar proportions in G0 (39/52) and G1 samples (55/75) (Supplementary Figure 1). These data suggest that the majority of individuals with SARS-CoV-2 specific Spike Pan Ig antibody responses also have SARS-CoV-2 specific neutralising antibody responses against Spike protein.

### SARS-CoV-2 specific T-cell response

T-cell responses were measured by IFN-γ ELISpot assays using pools of overlapping 15-18mer peptides spanning S (S1, S2), N, membrane (M) and envelope (E) proteins as well as non-structural regions of ORF1, and accessory proteins (ORF3, ORF6, ORF7 and ORF8) in cases, or peptides spanning S1, S2, M, N, NSP3B, NSP12A, NSP12B, NSP13 and NSP15+18 for control groups. In total, peripheral blood SARS-CoV-2-specific T-cell responses from 109 cases (29 G0 female, 52 G1 female, 15 G0 male and 13 G1 male participants), 92 matched controls (33 female G0, 34 female G1, 11 male G0, 14 male G1) and 82 random controls (33 female G0, 49 male G1) were analysed (Supplementary Figures 2-4). For all groups, SARS-CoV-2-specific IFN-γ production is summarised in Supplementary Table 2.

T-cell responses specific for S and N were higher in cases compared to matched and random control groups ( Figures 2D–E). Similarly, the total SARS-CoV-2-specific T-cell response assessed as the sum of responses to peptide pools spanning S, M, N and the non-structural proteins (NSP3B, NSP12A, NSP12B, NSP13 and NSP15-16) was higher in cases compared to control groups ( Figure 2F and Supplementary Figure 4). Within the case group (n = 109), T-cell responses of the greatest magnitude were observed against S1, S2, M, N and NSP3B. N and S proteins were also more commonly recognized by T-cells across individuals, with respectively 92.6% (101/109) and 91.7% (100/109) of cases demonstrating antigen-specific IFN-γ production in response to peptide pools spanning these proteins. This was followed by over 75% of cases displaying T-cell responses to M, and respectively 61% and 55% of participants displaying responses to NSP12B and NSP3B.

SARS-CoV-2 T-cell responses did not show strong evidence of difference between females and males nor between G0 and G1 in cases (Supplementary Figure 2) and controls (Supplementary Figure 3). The only exception to this trend was the higher T-cell response to M and ORF7 observed in female G0 cases compared to their G1 counterpart. In summary, these data demonstrate the generation of memory T-cell responses targeting all SARS-CoV-2 proteins in the case group, with a preferential recognition of S and N (>90% of cases).

Overall, individuals who have a confirmed SARS-CoV-2 infection either by a positive LFT serology test and/or PCR test have greater levels of anti-S and anti-N binding antibodies, a higher frequency of T-cells targeting S, M and N peptides and of total SARS-CoV-2-specific T-cells, and a greater level of neutralising antibodies as compared to the matched and random controls ( Figure 2, Supplementary Figures 1–4).

### Correlation between the immune memory variables

**Figure 3.**
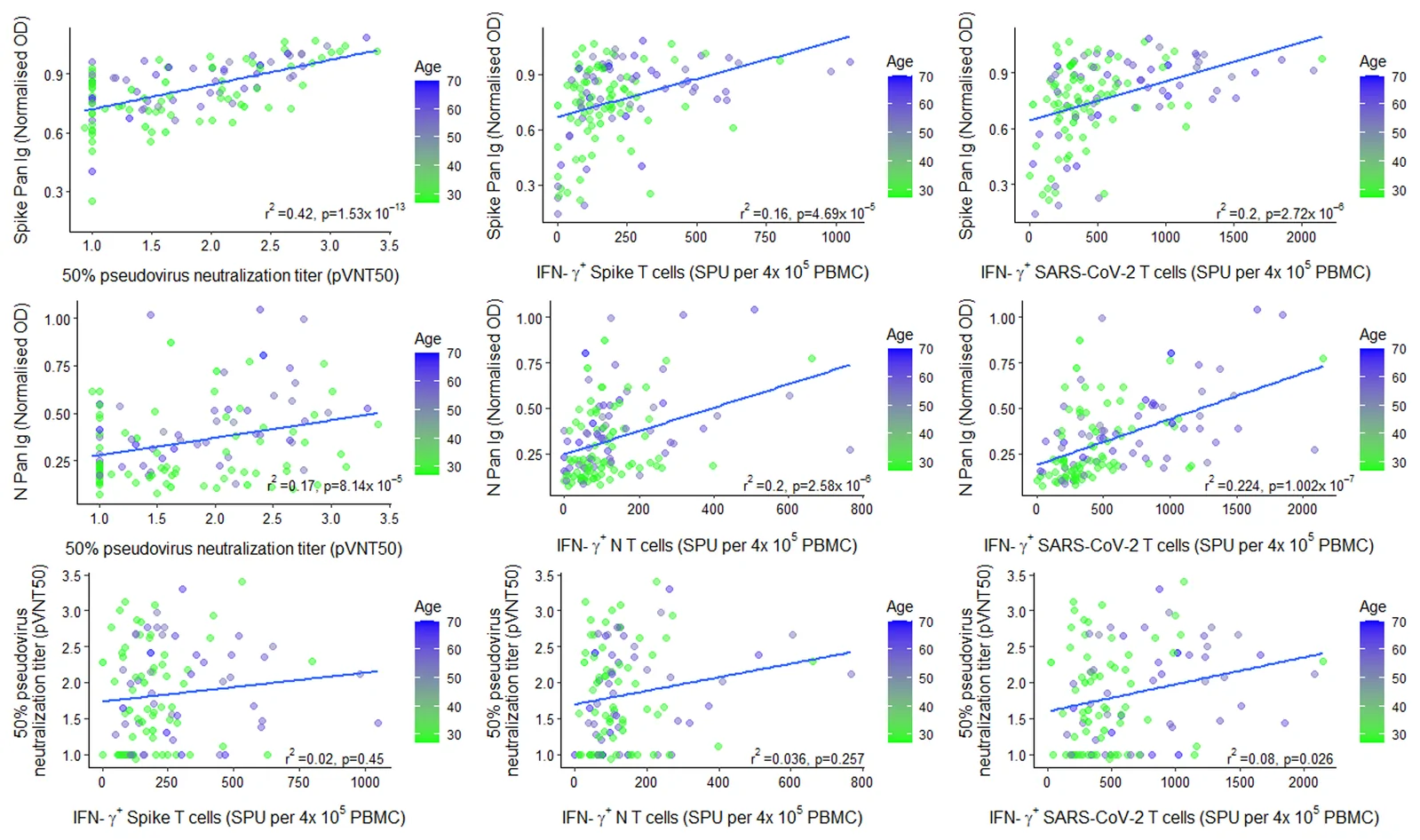
Correlation between different components of the immune response to SARS-CoV-2. **First row:** correlations between SARS-CoV-2-specific antibody response to anti-Spike Pan Ig antibody plotted against neutralisation of pseudotyped VSV by serum; SARS-CoV-2-specific IFN-γ Spike T-cell response; SARS-CoV-2-specific IFN-γ T-cell response to summed SARS-CoV-2 peptide pools spanning Spike, M, N proteins and non-structural proteins (NSP3b, NSP12A, NSP12B, NSP13 and NSP15-16). **Second row:** correlations between SARS-CoV-2-specific antibody response to anti-Nucleocapsid Pan Ig antibody plotted against neutralisation of pseudotyped VSV by serum; SARS-CoV-2-specific IFN-γ nucleocaspid T-cell response; SARS-CoV-2-specific IFN-γ T-cell response to summed SARS-CoV-2 peptide pools spanning Spike, M, N proteins and non-structural proteins (NSP3b, NSP12A, NSP12B, NSP13 and NSP15-16). **Third row:** correlations between neutralisation of pseudotyped VSV by serum plotted against SARS-CoV-2-specific IFN-γ Spike T-cell response; SARS-CoV-2-specific IFN-γ nucleocapsid T-cell response; SARS-CoV-2-specific IFN-γ T-cell response to summed SARS-CoV-2 peptide pools spanning Spike, M, N proteins and non-structural proteins (NSP3b, NSP12A, NSP12B, NSP13 and NSP15-16). The correlation coefficient (r ^2^) present alongside its p value calculated using linear regression.

From this point on, all analyses were conducted on analysis cases only (see methods). There was a positive correlation between anti-S binding antibody levels and neutralising antibody titres (p=1.53x10 ^-13^, r ^2^ = 0.42), IFN-γ producing S-specific T-cells (p=4.69x10 ^-5^, r ^2^ = 0.16) and IFN-γ producing SARS-CoV-2 specific T-cells (p=2.72x10 ^-6^, r ^2^ = 0.2). In addition, we observed a positive correlation between anti-N binding antibody levels and neutralising antibodies (p=8.14x10 ^-5^, r ^2^ = 0.17), IFN-γ producing N-specific T-cells (p=2.58x10 ^-6^, r ^2^ = 0.2) and IFN-γ producing SARS-CoV-2 specific T-cells (p=1.002x10 ^-7^, r ^2^ = 0.2). Neutralising antibody titres correlate to a lesser degree with IFN-γ producing SARS-CoV-2 specific T-cells (p=0.026, r ^2^ = 0.08) ( Figure 3). Whilst modest, the positive correlation detected between antibody levels and IFN-γ producing T-cells suggests that antibodies, which are easy to measure at a population level, may help to understand the magnitude of the COVID-19 specific T-cell response at a population level when measured within 9/10 months of infection.

### Waning of memory immune response

**Figure 4.**
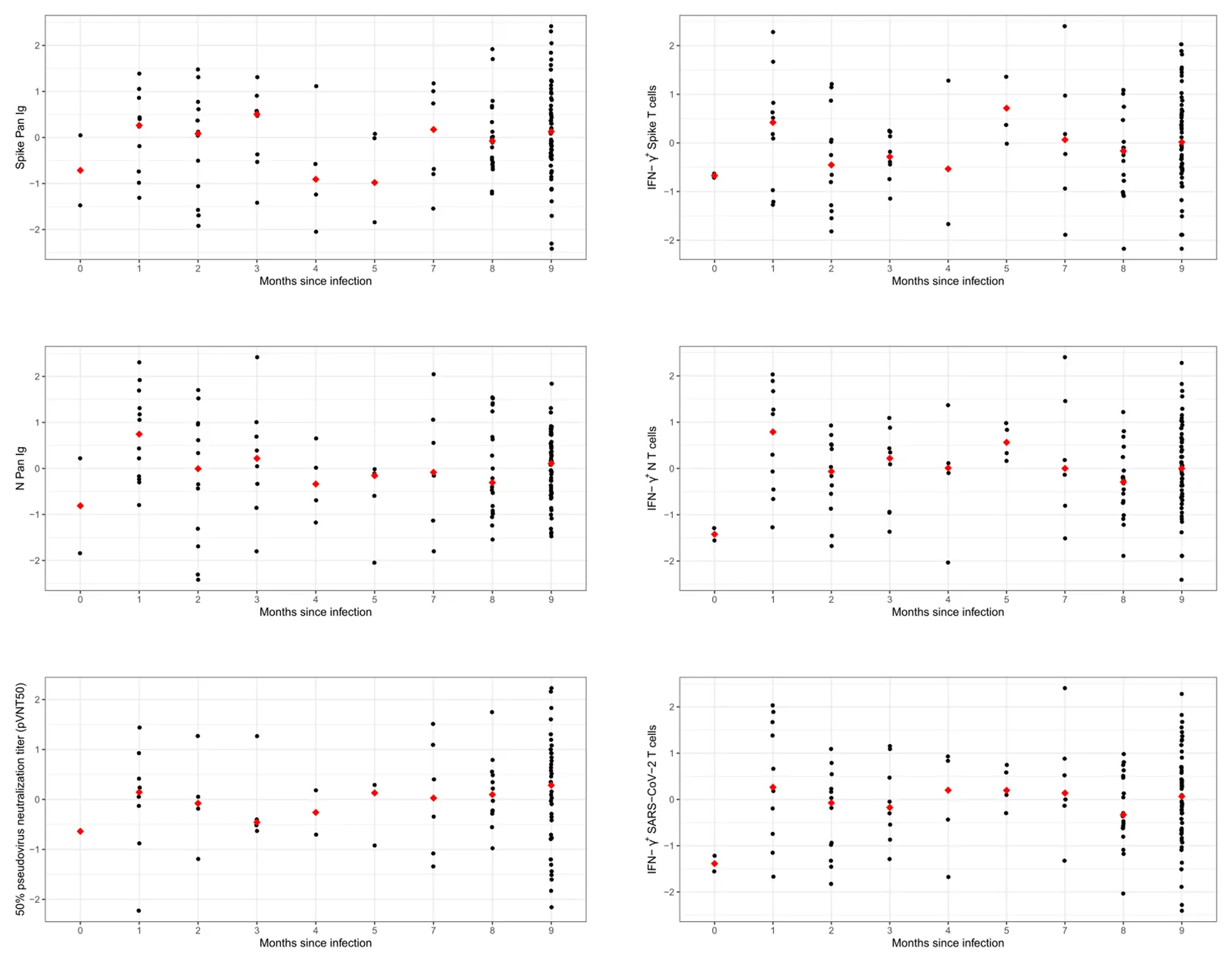
Immune variables and response and time from infection. Months since infection were determined from regular questionnaires completed during the pandemic. The immune variables were inverse normal rank transformed and are expressed as standard deviations. Graphs show the magnitude of immune variable over 9 months since infection.

To examine whether the magnitude of the immune memory response at the time of measurement in December 2020 is related to a waning in immune response over time since infection, we plotted each immune variable measured against time since infection (as derived using the month of infection by self-reported symptoms). In this work, nine months since infection corresponds to March 2020, when COVID-19 infection incidence was high, hence the clustering of cases around this time point. There was no clear signal of waning for any of the antigen-specific antibody or T-cell responses measured ( Figure 4). Consequently, we did not adjust for timing of infection in our subsequent analyses.

### Associations between symptom severity and immune memory

**Figure 5.**
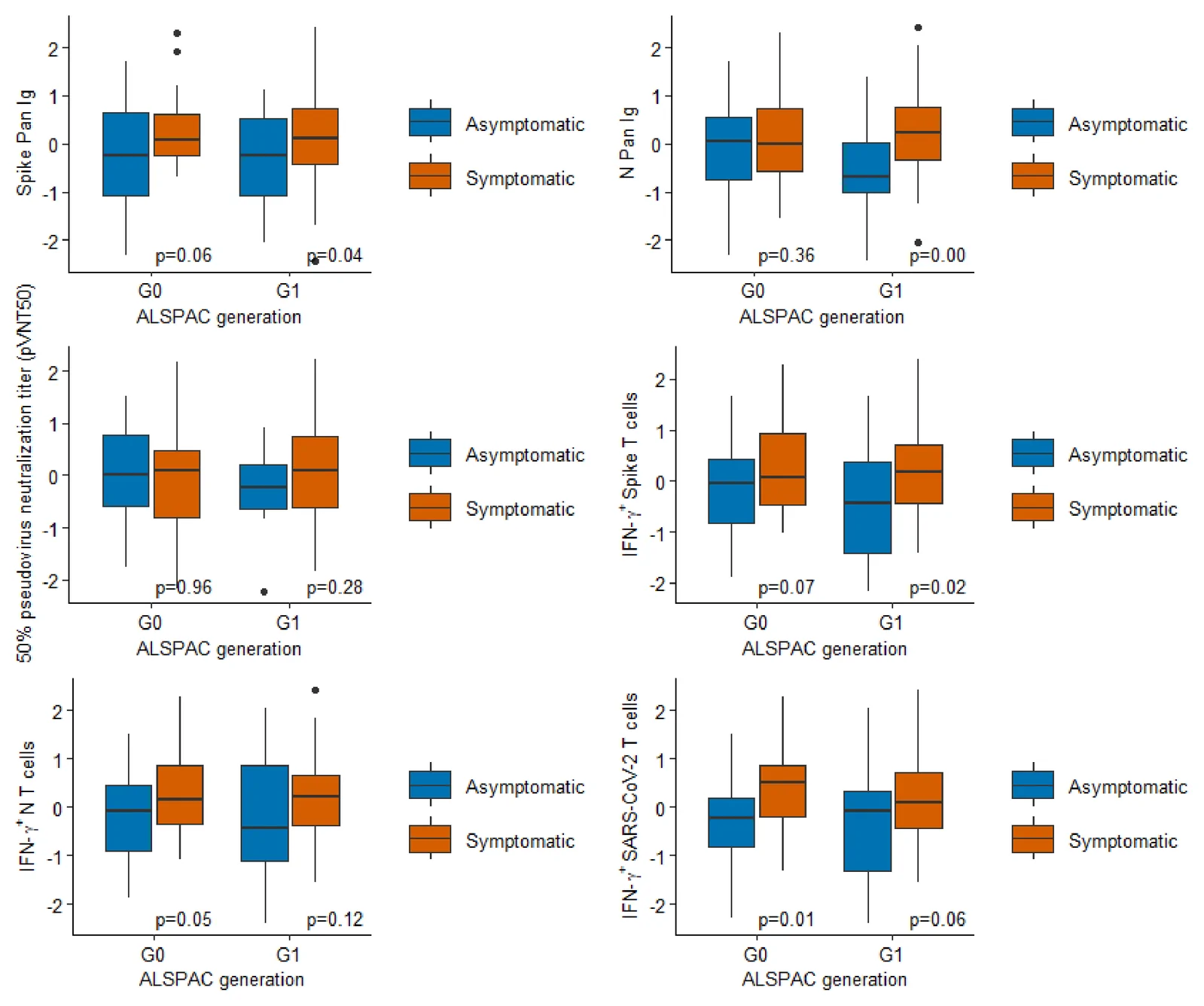
Immune variables and response by generation and symptom profile. Asymptomatic and symptomatic cases were determined using the Menni algorithm. The immune variables were inverse normal rank transformed and are expressed as standard deviations. P values from T tests comparing asymptomatic versus symptomatic cases within each generation.

Determining symptomatic and asymptomatic cases using the Menni algorithm ^30^, there was a trend towards an immune memory response of greater magnitude in the symptomatic compared to the asymptomatic cases of SARS-CoV-2, observed across the majority of immune variables and across both G0 and G1 generations ( Figure 5, Supplementary Figure 5). For example, for G0, the mean standardized IFN-γ producing SARS-CoV-2 specific T-cell value was greater in the symptomatic than asymptomatic cases (p=0.01, 0.43 compared with -0.27 SD); and for G1, the mean standardized anti-N binding antibody level was also greater in the symptomatic cases (p=0.00, 0.25 versus –0.53 SD).

**Figure 6.**
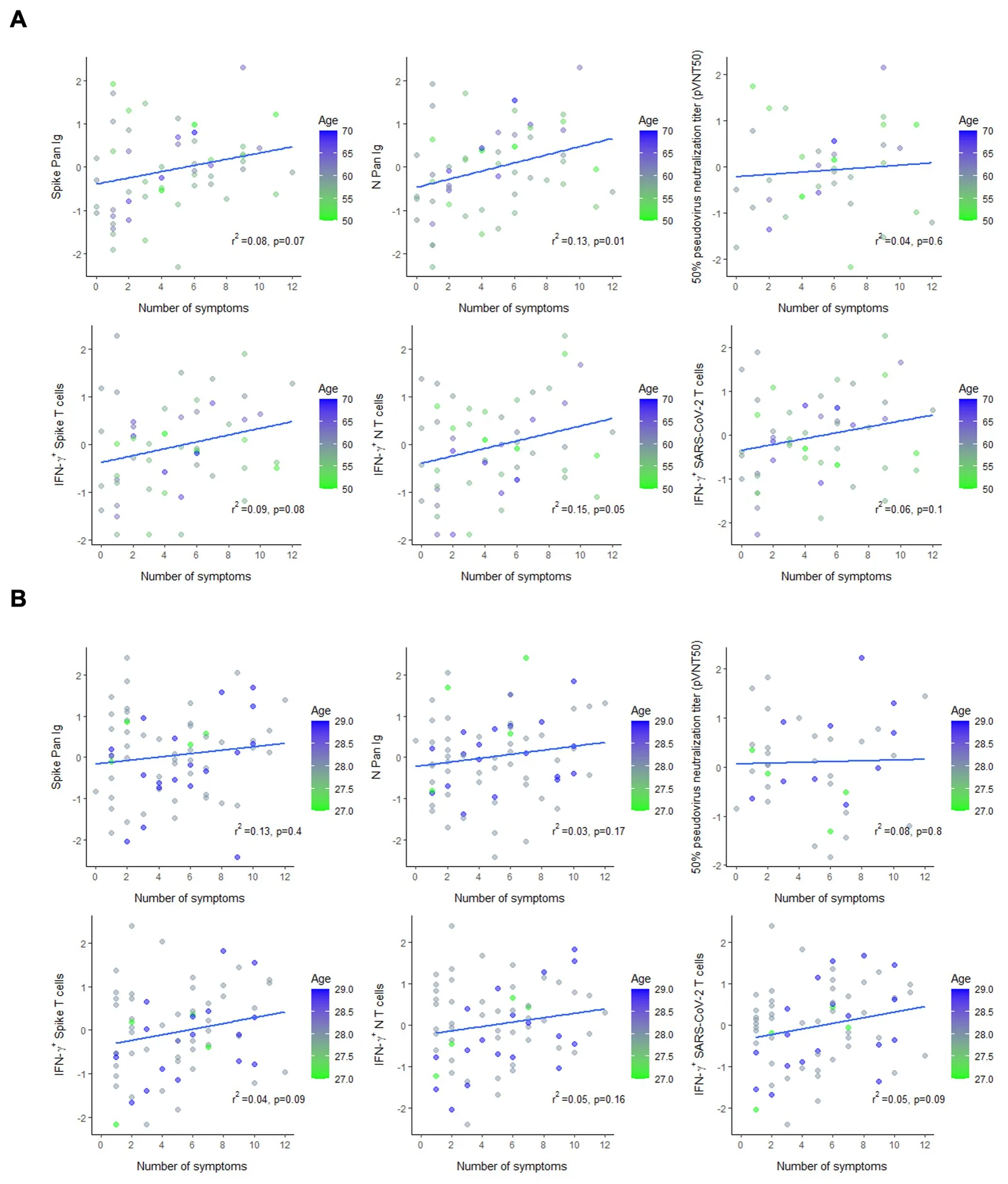
Correlation between magnitude of the immune response and symptoms. Number of symptoms were determined from regular questionnaires completed during the pandemic. Correlation plots of SARS-CoV-2-specific antibody response to anti-Spike and anti-N Pan Ig antibodies, neutralisation of pseudotyped VSV by serum and SARS-CoV-2-specific IFN-γ spike T-cell response, SARS-CoV-2-specific IFN-γ nucleocapsid T-cell response; SARS-CoV-2-specific IFN-γ T-cell response against number of symptoms experienced by an individual following SARS-CoV-2 infection, plotted by generation ( **A**) G0 ( **B**) G1. The immune variables were inverse normal rank transformed and are expressed as standard deviations. The correlation coefficient (r ^2^) present alongside its p value calculated using linear regression.

**Figure 7.**
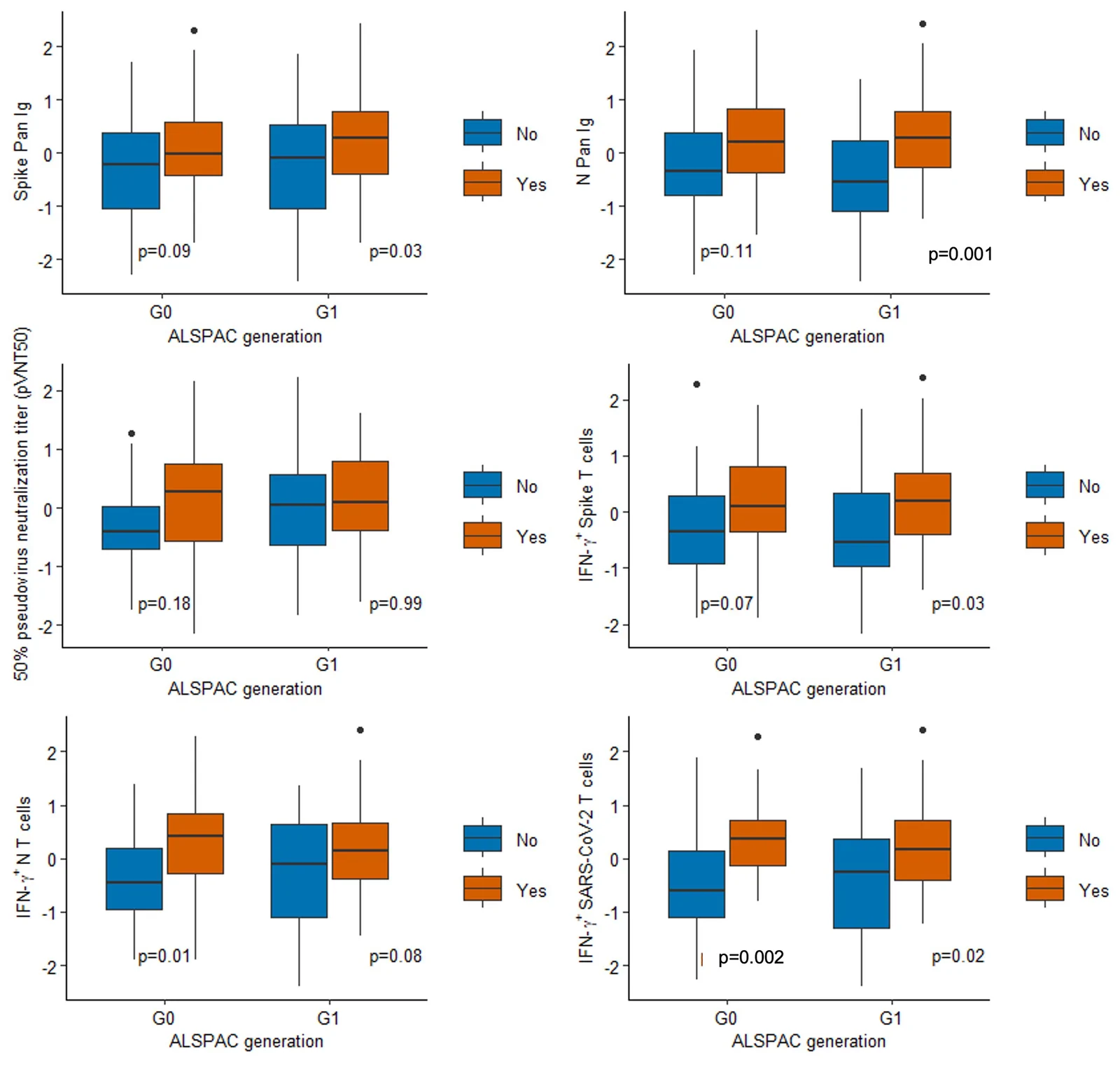
Association between loss of taste and/or smell immune response. Cases with loss of taste and/or smell were determined from regular questionnaires completed during the pandemic. The immune variables were inverse normal rank transformed and are expressed as standard deviations. P values from T tests comparing cases with loss of taste and/or smell within each generation.

Given that we had access to data on symptoms by month, we also derived an alternative definition of symptom severity, namely an estimate of the *number* of symptoms associated with a COVID-19 infection, as detailed in the methods. Using this alternative definition, we observed a positive correlation between the number of symptoms and the magnitude of the immune variables measured in the older G0 cohort, for anti-N binding antibodies (p=0.01, r ^2^ =0.13) and IFN-γ producing N-specific T-cells (p=0.05, r ^2^ =0.15) ( Figure 6). Furthermore, focusing on the specific symptom of loss of taste and/or smell, which at the start of the pandemic was identified as a distinct symptom of SARS-CoV-2 infection, we observed that those individuals with this particular symptom tended to have a greater magnitude of IFN-γ producing N-specific T-cells in the G0 generation (p=0.01) and IFN-γ producing SARS-CoV-2 specific T-cells (p=0.00 for G0; p=0.02 for G1) than those who did not experience loss of taste or smell ( Figure 7).

### Impact of prior health related measures on the SARS-CoV-2 memory response

**Figure 8.**
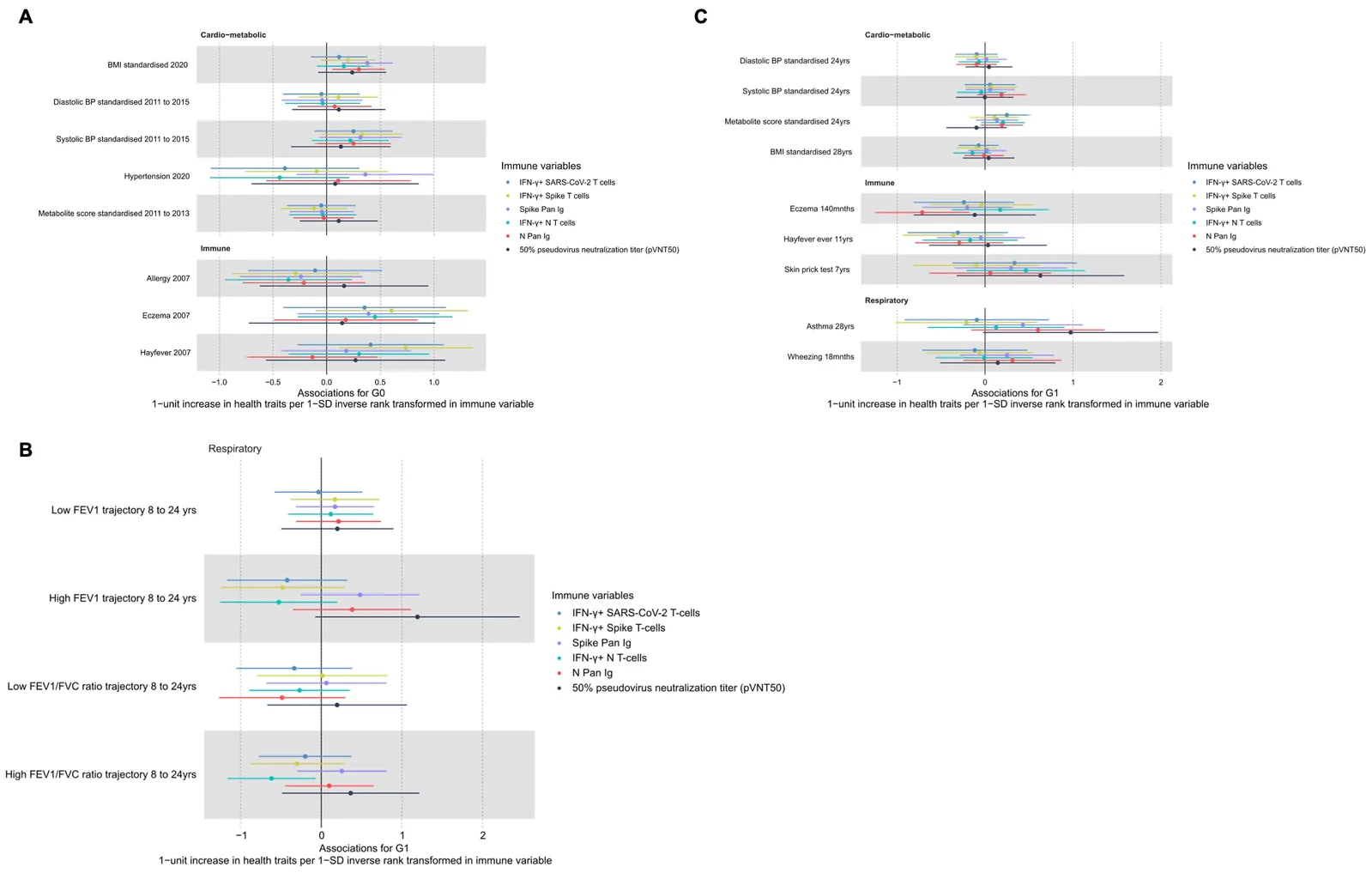
Association between immune response and life course factors. **A** analysis for the G0 generation and **B** for the G1 generation. Prior health factors were measured at various points prior to the pandemic. The immune variables were inverse normal rank transformed and are expressed as standard deviations. Linear regression was undertaken to investigate the relationship between health factors and memory immune response to SARS-CoV-2. Betas are expressed per unit increase in health trait for a 1-SD change in inverse rank transformed immune variable. **C** shows G1 respiratory variables: forced expiratory volume during the first second (FEV1) trajectory, below average trajectory versus average; FEV1 trajectory, persistently high trajectory versus average; ratio of FEV1 to forced vital capacity (FVC) trajectory, below average trajectory versus average; and FEV1/FVC trajectory, persistently high trajectory versus average.

There were insufficient numbers of participants with diabetes to be able to carry out analyses; similarly for hypertension in G1. There was a trend towards a higher systolic blood pressure prior to the pandemic associating with higher immune memory responses in G0 ( Figure 8A). BMI was not associated with the magnitude of the immune memory response in the G1 generation ( Figure 8B), however in the older G0 generation, higher BMI was associated with an immune memory response of greater magnitude seen particularly with anti-S binding antibodies (β = 0.38 per unit increase in health trait for a 1-SD change in inverse rank transformed immune variable, 95% CI: 0.14, 0.62, p=0.003) and anti-N binding antibodies (β = 0.30 per unit increase in health trait for a 1-SD change in inverse rank transformed immune variable, 95% CI: 0.05, 0.55, p=0.02) ( Figures 8A and 8B). Both associations persisted when taking into account symptoms.

A decreased expression of angiotensin-converting enzyme 2 (ACE2) has been reported in individuals who have asthma together with high levels of allergic sensitisation ^33^. In line with this, atopic disease has been associated with decreased odds of developing COVID-19 ^34^. We looked at the presence of eczema, hay fever and allergy within G0 and eczema at 140 months, hay fever at age 11, allergic sensitivity via a skin prick test performed at age 7 within G1. The trends of association with the immune variables are in opposite directions in the different generations suggesting that childhood eczema, hayfever and allergy may affect the development of memory immune response to SARS-CoV-2 differently compared to adulthood eczema, hayfever and allergy. Within G1, there was a lower memory response (anti-N antibodies) for those who had eczema at 140 months (β = -0.7 per unit decrease in health trait for a 1-SD change in inverse rank transformed immune variable, 95% CI: -1.26, -0.17, p=0.01). However, eczema measured in adulthood for the G0 generation no longer showed an association with immune response. For G0, there was a positive association between the presence of hay fever in adulthood and IFN-γ producing Spike T-cells (β=0.74 per unit increase in health trait for a 1-SD change in inverse rank transformed immune variable, 95% CI:0.09, 1.38, p=0.03) ( Figure 8). These associations persist when the model is adjusted for symptoms and when considering the wider set of immune variables, we see associations of eczema in G0 and hay fever in G1 with T-cell responses targeting certain proteins (Supplementary File), again associations persisting after the addition of symptoms.

A multi-metabolic biomarker score, consisting of 25 proteins, fatty acids, amino acids, and lipids has previously been associated with of being a hospitalised COVID-19 in UK Biobank ^35^. We investigated whether this score was associated with the magnitude of the memory immune response in participants in this study. In the older generation (G0), there is no association. However, in the younger generation (G1), a trend towards a positive association with all the immune variables apart from neutralising antibodies was observed ( Figure 8).

For the older G0 cohort, we did not have sufficient individuals with asthma to undertake the analysis. No association was observed with any of the immune markers for the G1 participants who reported asthma in the COVID-1 questionnaire. However, we were able to consider more nuanced measures of respiratory health collected across the life course for this younger cohort. When comparing immune markers for those with below average or persistently high forced expiratory volume (FEV1) and Tiffeneau-Pinelli index (FEV1/FVC ratio) trajectories, compiled from repeat spirometry from childhood into early adulthood, against those for individuals with an average trajectory ^36^, we found that a higher FEV1/FVC ratio trajectory was negatively associated with IFN-γ producing N-specific T-cells (p=0.03) ( Figure 8C), and this relationship persisted when adjusted for symptoms. The proportion of G1 participants presenting with wheezing, or wheezing with whistling (measured approximately annually and referring to symptoms in the last 12 months) ^37^, peaked at time point 6–18 months, so we also chose to look at this time point in more detail, but found no evidence for associations between the incidence of wheezing and immune response to SARS-CoV-2 infection ( Figure 8B).

**Figure 9.**
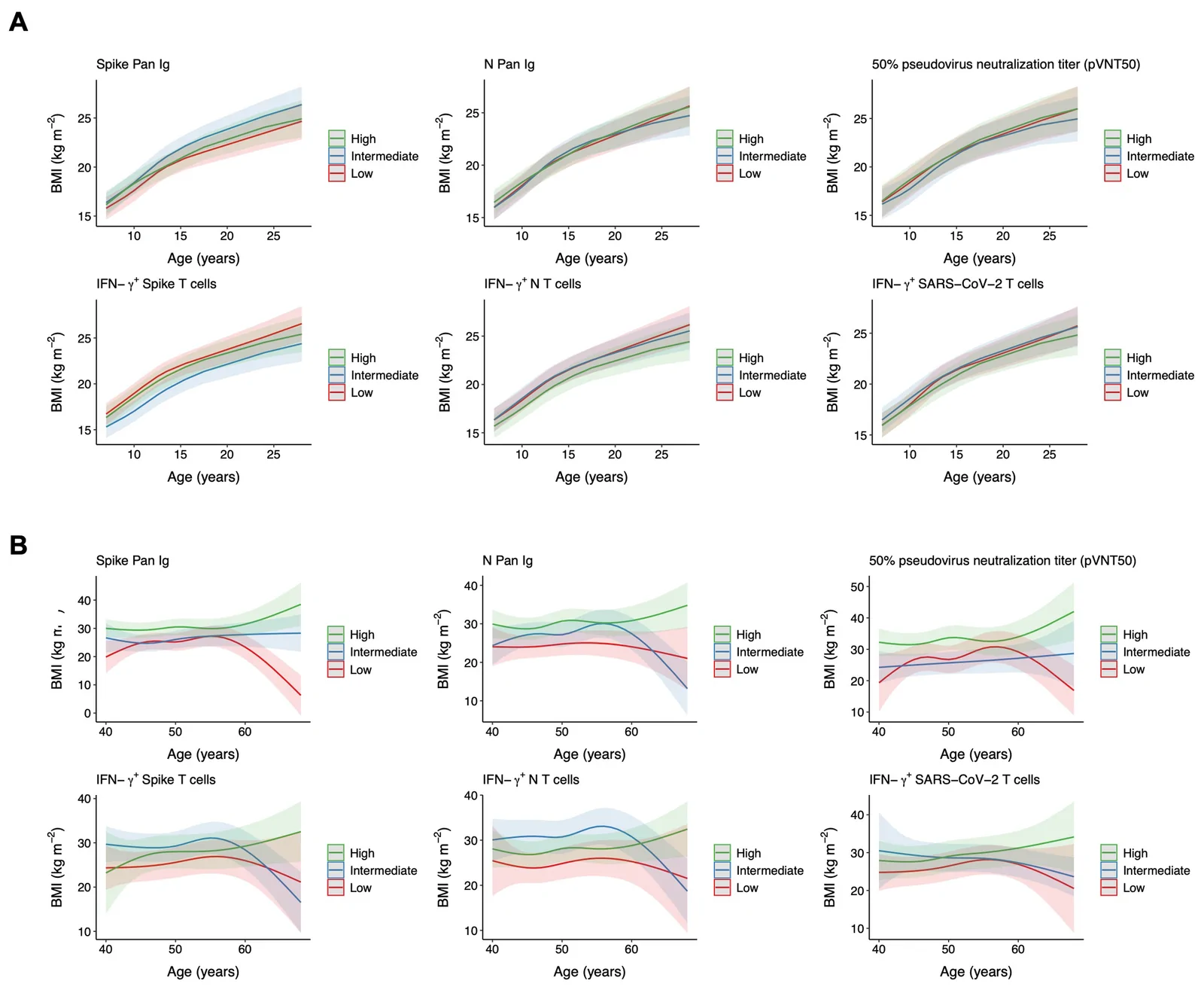
Predicted mean trajectories of BMI by magnitude of memory immune response. Predicted mean trajectories of BMI by magnitude of memory immune response. Predicted means were obtained using natural cubic spline linear mixed-effects trajectory models. ( **A**) shows the predicted mean trajectories in BMI from childhood to adulthood. ( **B**) Predicted mean trajectories of BMI by magnitude of memory immune response in the 20 years prior to the pandemic in G0.

As a longitudinal study, ALSPAC provides the opportunity to examine how changes in BMI over time might impact on the SARS-CoV-2 memory response. Time series analyses were conducted to examine the association between BMI trajectory and the immune memory response to SARS-CoV-2 infection. In the younger generation (G1), no association between BMI over time during childhood and adolescence and the magnitude of memory immune response established following SARS-CoV-2 infection was detected ( Figure 9A and Supplementary Figure 6A). In the older generation (G0), examining BMI over the 20 years prior to infection with SARS-CoV-2, those with a higher antibody immune response had a sustained high BMI over that time period ( Figure 9B and Supplementary Figure 6B).

## Discussion

In this study we investigated the memory immune response following natural SARS-CoV-2 infection in a non-hospitalised general population, without prior vaccination, with an age range of 27–70 years. This unique study examined the memory immune response in mild and asymptomatic infection. Specifically, we quantified binding antibody responses through the measurement of both anti-S and anti-N antibodies, functionally relevant neutralisation antibody titres and the T-cell IFN-γ response to proteins encoded by SARS-CoV-2. We observed a memory immune response following SARS-CoV-2 infection with a positive correlation between all immune variables measured in individuals with mild or asymptomatic infection. The magnitude of these memory responses was not related to age and sex in this collection. An advantage of this study is the longitudinal and deeply phenotyped nature of the ALSPAC cohort providing the opportunity to examine the impact of prior health factors to SARS-CoV-2 infection on the immunological response to infection. To that end we presented a limited exemplar set of association analyses, informed by risk factors for severe infection, to illustrate how relationships between life course factors and immune response in mild and asymptomatic disease can be characterised using well-placed cohort studies.

Adaptive immune responses are important for the control and clearance of viral infections, with development of long-term immunological memory providing ongoing protection against SARS-CoV-2 ^5^. Although linked in important ways, the protective roles of humoral and cellular immunity can differ depending on the viral infection as well as other factors. Therefore, it is important to assess both antibody responses as well as antigen-specific T-cells in response to a SARS-CoV-2 infection. Although there has been no correlation found between viral shedding and symptoms ^38^, we recognise that the magnitude of the viral load ^39^ alongside the innate immune response ^40^ both play a role in the subsequent adaptive and therefore immune memory response. As there was no means of measuring either parameter in this study, interpretation of the results has been made independently of these factors. However, within this study we interrogate what factors are associated with variation in the memory immune response.

At a population level, antibodies benefit from ease of detection and assessment whereas cellular responses are more difficult to study and impractical to measure at scale. Serological antibody tests are being used as a marker of previous infection with SARS-CoV-2 ^41^. Our work suggests that there is a positive correlation between the binding antibody response and both neutralising antibodies and T-cell responses in agreement with previously reported findings following natural infection of SARS-CoV-2 ^7, 32, 42– 44^. Therefore, antibodies in a population may be a useful surrogate marker of T-cell responses and assessing the adaptive immune response to SARS-CoV-2. We also found in this study that within individuals who had previously been infected with SARS-CoV-2, antibodies directed to the S protein were observed more frequently than to the N protein (95/114 and 41/114 respectively), which has implications for studies using solely the anti-N antibody to define cases.

A greater magnitude in T-cell immune response has been shown in symptomatic versus asymptomatic non-hospitalised COVID-19 cases 6 months post-infection ^42^. Our data corroborates this and expands it to include humoral immune responses using the Menni algorithm ^45^ to define symptomatic and asymptomatic cases. As another way of examining symptom severity, we investigated number of symptoms and loss of taste and smell and found that a greater number of COVID-19 symptoms and loss of taste and smell were associated with a greater magnitude of memory immune response. Indeed, we saw signs that the Menni algorithm did not identify all infected individuals, especially those who had not lost their sense of smell or taste, and hence our results based on the Menni algorithm are likely to be conservative. It is important to stress that our study was performed in mild cases of COVID-19 and more severe disease has resulted in memory immune responses of greater magnitude ^11, 46^. Our measures of symptom severity were derived from questionnaire data and hence may suffer from under or mis-reporting. However, these results are in line with previous works showing higher IgG antibody response ^47^ and T-cell responses ^7^ in severe as compared to mild disease.

In investigating health factors in relation to the magnitude of memory immune response, there is a balance between an immune response being established sufficiently to clear the virus, limiting the extent of the disease, and an overactive immune response which can result in severe disease. During the early phases of infection, the timing and strength of the initial T-cell responses could be critical in determining this balance ^7^. The majority of individuals who recover from COVID-19 display robust T-cell responses and interestingly there appears to be some evidence of ‘cellular sensitization without seroconversion’ whereby individuals exposed to SARS-CoV-2 display virus-specific cellular responses without evidence of virus-specific antibodies ^8, 48, 49^.

We restricted analysis to cases in the original study design and individuals who were originally recruited as controls but had anti-S and anti-N antibodies by ELISA. This potentially highlights a difference in assay performance between measuring antibodies by LFT and ELISA. This enabled us to capture in our ‘analysis cases’ individuals who were infected with SARS-CoV-2 between time of recruitment and the clinic as defined by ELISA antibody measurement. Due to restricting to a case only study, this will elicit index-event bias/collider bias in the study design ^50^ and the results need to be interpreted in light of this. For example, eczema has been shown to be protective for susceptibility to COVID-19 infection. Therefore, the individuals in our study sample are a sub-sample and therefore the comparison in eczema as a risk factor is difficult.

One of the major advantages of this study is the fact that the individuals recruited to this study were all participants within the ALSPAC cohort. Firstly, this provides an opportunity to examine the immune response to SARS-CoV-2 in two generations of individuals – the younger generation (G1) with an average age of 28 and the older generation (G0) with an average age of 58. Age is a known risk factor for COVID-19 severity. Notably, we found that age did not associate strongly with the immune response in mild COVID-19, consistent with previous work in relation to the antibody response ^47^. We also observed some association between age and T-cell responses, consistent with previous results showing increased T-cell response for individuals aged 60 and over ^51^ and lower T-cell responses in children under 13 years ^52^.

Importantly, the longitudinal nature of ALSPAC provides the opportunity to look at health factors prior to SARS-CoV-2 infection and examine the impact on the immunological response to infection. This also allows the investigation of whether health factors known to influence severe disease also influence mild or asymptomatic infection, and whether any associations between health factors and immune memory response are attenuated when symptom severity is included as a covariate in the model.

Obesity has been shown as a risk factor for COVID-19 severity ^53^ and leads to increased inflammation. We showed that BMI is associated with an immune response of greater magnitude in the older generation following SARS-CoV-2 infection particularly for S and N specific T-cells and both binding and neutralising antibodies. This is consistent with the findings of a small study that showed that memory T-cells correlated with obesity in older cases of COVID-19 but not younger cases ^54^. It has been shown that convalescent COVID-19 patients with higher BMI have an increased titre of binding antibodies ^52, 55^ although this was not the case in a smaller sample size ^56^. Therefore, our findings are consistent with the observation that adiposity appears to influence the immune response.

In conclusion, we observed that memory immune responses to SARS-CoV-2 infection can be detected in mild cases of COVID-19 at least up to 9 months post-infection. Infection that resulted in symptoms elicited a memory immune response of greater magnitude, although variation in immune response was also seen in those who were asymptomatic or had few symptoms. A major strength of this study is that participants were embedded within the ALSPAC cohort, so health data collected for many years prior to the pandemic was available. This allowed for the investigation of prior health factors that modify the immune response in mild infection. Prior health factors such as BMI were shown to associate with the magnitude of the memory immune response to SARS-CoV-2. These results provide a novel understanding on how health factors prior to an infection may impact the ability to mount a memory immune response in mild infection.

## Ethical approval and consent

Ethical approval for the study was obtained from the ALSPAC Ethics and Law Committee and the Local Research Ethics Committees (NHS London, Camden and Kings Cross REC: 20/HRA/4854; 2020). Consent for biological samples has been collected in accordance with the Human Tissue Act (2004). Informed consent for the use of data collected via questionnaires and clinics was obtained from participants following the recommendations of the ALSPAC Ethics and Law Committee at the time. At age 18, study children were sent 'fair processing' materials describing ALSPAC’s intended use of their health and administrative records and were given clear means to consent or object via a written form. Data were not extracted for participants who objected, or who were not sent fair processing materials. Full details of the ALSPAC consent procedures are available on the study website ( https://www.bristol.ac.uk/alspac/researchers/research-ethics/).

## Data Availability

Please see the ALSPAC data management plan which describes the policy regarding data sharing ( http://www.bristol.ac.uk/alspac/researchers/data-access/documents/alspac-data-management-plan.pdf), which is by a system of managed open access. Data used for this submission will be made available on request to the Executive ( alspac-exec@bristol.ac.uk). OSF: SARS-CoV-2 memory response in non-hospitalised cases: immunology in the context of a population-based cohort study. https://doi.org/10.17605/OSF.IO/SHQAE ^29^ This project contains the following extended data: Supplementary material. PDF Data are available under the terms of the Creative Commons Attribution 4.0 International license (CC-BY 4.0). ALSPAC study data were collected and managed from participants at 22 years and onwards using REDCap electronic data capture tools hosted at the University of Bristol ^57^. REDCap (Research Electronic Data Capture) is a secure, web-based software platform designed to support data capture for research studies.

## References

[1] Hu B Guo H Zhou P : Characteristics of SARS-CoV-2 and COVID-19. *Nat Rev Microbiol.* 2021;19(3):141–154. 10.1038/s41579-020-00459-7 33024307 PMC7537588

[2] Coronaviridae Study Group of the International Committee on Taxonomy of Viruses: The species *severe acute respiratory syndrome-related coronavirus*: classifying 2019-nCoV and naming it SARS-CoV-2. *Nat Microbiol.* 2020;5(4):536–544. 10.1038/s41564-020-0695-z 32123347 PMC7095448

[3] Yang R Gui X Xiong Y : Comparison of clinical characteristics of patients with asymptomatic vs symptomatic coronavirus disease 2019 in Wuhan, China. *JAMA Netw Open.* 2020;3(5): e2010182. 10.1001/jamanetworkopen.2020.10182 32459353 PMC7254178

[4] Madden EA Diamond MS : Host cell-intrinsic innate immune recognition of SARS-CoV-2. *Curr Opin Virol.* 2022;52:30–38. 10.1016/j.coviro.2021.11.002 34814102 PMC8580835

[5] Sette A Crotty S : Adaptive immunity to SARS-CoV-2 and COVID-19. *Cell.* 2021;184(4):861–880. 10.1016/j.cell.2021.01.007 33497610 PMC7803150

[6] Nordström P Ballin M Nordström A : Risk of SARS-CoV-2 reinfection and COVID-19 hospitalisation in individuals with natural and hybrid immunity: a retrospective, total population cohort study in Sweden. *Lancet Infect Dis.* 2022;22(6):781–790. 10.1016/S1473-3099(22)00143-8 35366962 PMC8971363

[7] Peng Y Mentzer AJ Liu G : Broad and strong memory CD4 ^+^ and CD8 ^+^ T cells induced by SARS-CoV-2 in UK convalescent individuals following COVID-19. *Nat Immunol.* 2020;21(11):1336–1345. 10.1038/s41590-020-0782-6 32887977 PMC7611020

[8] Sekine T Perez-Potti A Rivera-Ballesteros O : Robust T cell immunity in convalescent individuals with asymptomatic or mild COVID-19. *Cell.* 2020;183(1):158–168.e114. 10.1016/j.cell.2020.08.017 32979941 PMC7427556

[9] Long QX Liu BZ Deng HJ : Antibody responses to SARS-CoV-2 in patients with COVID-19. *Nat Med.* 2020;26(6):845–848. 10.1038/s41591-020-0897-1 32350462

[10] Rydyznski Moderbacher C Ramirez SI Dan JM : Antigen-specific adaptive immunity to SARS-CoV-2 in acute COVID-19 and associations with age and disease severity. *Cell.* 2020;183(4):996–1012. e1019. 10.1016/j.cell.2020.09.038 33010815 PMC7494270

[11] Dan JM Mateus J Kato Y : Immunological memory to SARS-CoV-2 assessed for up to 8 months after infection. *Science.* 2021;371(6529): eabf4063. 10.1126/science.abf4063 33408181 PMC7919858

[12] Gallais F Velay A Nazon C : Intrafamilial exposure to SARS-CoV-2 associated with cellular immune response without seroconversion, France. *Emerg Infect Dis.* 2021;27(1):113–121. 10.3201/eid2701.203611 33261718 PMC7774579

[13] Swadling L Diniz MO Schmidt NM : Pre-existing polymerase-specific T cells expand in abortive seronegative SARS-CoV-2. *Nature.* 2022;601(7891):110–117. 10.1038/s41586-021-04186-8 34758478 PMC8732273

[14] Goenka A Halliday A Gregorova M : Young infants exhibit robust functional antibody responses and restrained IFN-γ production to SARS-CoV-2. *Cell Rep Med.* 2021;2(7): 100327. 10.1016/j.xcrm.2021.100327 34124701 PMC8188298

[15] Bange EM Han NA Wileyto P : CD8 ^+^ T cells contribute to survival in patients with COVID-19 and hematologic cancer. *Nat Med.* 2021;27(7):1280–1289. 10.1038/s41591-021-01386-7 34017137 PMC8291091

[16] McMahan K Yu J Mercado NB : Correlates of protection against SARS-CoV-2 in rhesus macaques. *Nature.* 2021;590(7847):630–634. 10.1038/s41586-020-03041-6 33276369 PMC7906955

[17] Beaney T Neves AL Alboksmaty A : Trends and associated factors for COVID-19 hospitalisation and fatality risk in 2.3 million adults in England. *Nat Commun.* 2022;13(1): 2356. 10.1038/s41467-022-29880-7 35487905 PMC9054846

[18] Huang C Wang Y Li X : Clinical features of patients infected with 2019 novel coronavirus in Wuhan, China. *Lancet.* 2020;395(10223):497–506. 10.1016/S0140-6736(20)30183-5 31986264 PMC7159299

[19] Boyd A Golding J Macleod J : Cohort profile: the 'children of the 90s'--the index offspring of the Avon Longitudinal Study of Parents and Children. *Int J Epidemiol.* 2013;42(1):111–127. 10.1093/ije/dys064 22507743 PMC3600618

[20] Fraser A Macdonald-Wallis C Tilling K : Cohort profile: the Avon Longitudinal Study of Parents and Children: ALSPAC mothers cohort. *Int J Epidemiol.* 2013;42(1):97–110. 10.1093/ije/dys066 22507742 PMC3600619

[21] Northstone K Lewcock M Groom A : The Avon Longitudinal Study of Parents and Children (ALSPAC): an update on the enrolled sample of index children in 2019 [version 1; peer review: 2 approved]. *Wellcome Open Res.* 2019;4:51. 10.12688/wellcomeopenres.15132.1 31020050 PMC6464058

[22] Northstone K Shlomo YB Teyhan A : The Avon Longitudinal Study of Parents and Children ALSPAC G0 partners: a cohort profile [version 1; peer review: 1 approved with reservations]. *Wellcome Open Res.* 2023;8:37. 10.12688/wellcomeopenres.18782.1

[23] Northstone K Howarth S Smith D : The Avon Longitudinal Study of Parents and Children - a resource for COVID-19 research: questionnaire data capture April-May 2020 [version 2; peer review: 2 approved]. *Wellcome Open Res.* 2020;5:127. 10.12688/wellcomeopenres.16020.2 33628949 PMC7883314

[24] Northstone K Smith D Bowring C : The Avon Longitudinal Study of Parents and Children - a resource for COVID-19 research: questionnaire data capture May-July 2020 [version 2; peer review: 2 approved]. *Wellcome Open Res.* 2020;5:210. 10.12688/wellcomeopenres.16225.2 32995559 PMC7512032

[25] Northstone K Smith D Bowring C : The Avon Longitudinal Study of Parents and Children - a resource for COVID-19 research: home-based antibody testing results, October 2020. An emphasis on self-screening at a population level [version 2; peer review: 1 approved, 2 approved with reservations]. *Wellcome Open Res.* 2021;6:34. 10.12688/wellcomeopenres.16616.2 34622014 PMC8453314

[26] Smith D Bowring C Wells N : The Avon Longitudinal Study of Parents and Children - a resource for COVID-19 research: questionnaire data capture November 2020 – March 2021 [version 2; peer review: 2 approved]. *Wellcome Open Res.* 2021;6:155. 10.12688/wellcomeopenres.16950.2 34796274 PMC8591520

[27] Major-Smith D Matthews S Breeze T : The Avon Longitudinal Study of Parents and Children - a resource for COVID-19 research: antibody testing results, April – June 2021 [version 2; peer review: 2 approved]. *Wellcome Open Res.* 2022;6:283. 10.12688/wellcomeopenres.17294.2 35028425 PMC8738971

[28] Statista: Cumulative number of Coronavirus (COVID-19) cases in the United Kingdom (UK).2023. Reference Source

[29] Mitchell R : SARS-CoV-2 memory response in non-hospitalised cases: immunology in the context of a population-based cohort study. [Dataset]. August 6,2024. 10.17605/OSF.IO/SHQAE

[30] Menni C Valdes AM Freidin MB : Real-time tracking of self-reported symptoms to predict potential COVID-19. *Nat Med.* 2020;26(7):1037–1040. 10.1038/s41591-020-0916-2 32393804 PMC7751267

[31] Cervia C Zurbuchen Y Taeschler P : Immunoglobulin signature predicts risk of post-acute COVID-19 syndrome. *Nat Commun.* 2022;13(1): 446. 10.1038/s41467-021-27797-1 35078982 PMC8789854

[32] Halliday A Long AE Baum HE : Development and evaluation of low-volume tests to detect and characterize antibodies to SARS-CoV-2. *Front Immunol.* 2022;13: 968317. 10.3389/fimmu.2022.968317 36439154 PMC9682908

[33] Jackson DJ Busse WW Bacharier LB : Association of respiratory allergy, asthma, and expression of the SARS-CoV-2 receptor *ACE2*. *J Allergy Clin Immunol.* 2020;146(1):203–206. e3. 10.1016/j.jaci.2020.04.009 32333915 PMC7175851

[34] Holt H Talaei M Greenig M : Risk factors for developing COVID-19: a population-based longitudinal study (COVIDENCE UK). *Thorax.* 2022;77(9):900–912. 10.1136/thoraxjnl-2021-217487 34848555

[35] Julkunen H Cichonska A Slagboom PE : Metabolic biomarker profiling for identification of susceptibility to severe pneumonia and COVID-19 in the general population. *eLife.* 2021;10: e63033. 10.7554/eLife.63033 33942721 PMC8172246

[36] Belgrave DCM Granell R Turner SW : Lung function trajectories from pre-school age to adulthood and their associations with early life factors: a retrospective analysis of three population-based birth cohort studies. *Lancet Respir Med.* 2018;6(7):526–534. 10.1016/S2213-2600(18)30099-7 29628377

[37] Granell R Henderson AJ Sterne JA : Associations of wheezing phenotypes with late asthma outcomes in the Avon Longitudinal Study of Parents and Children: a population-based birth cohort. *J Allergy Clin Immunol.* 2016;138(4):1060–1070. e11. 10.1016/j.jaci.2016.01.046 27106203 PMC5052126

[38] Killingley B Mann AJ Kalinova M : Safety, tolerability and viral kinetics during SARS-CoV-2 human challenge in young adults. *Nat Med.* 2022;28(5):1031–1041. 10.1038/s41591-022-01780-9 35361992

[39] Pujadas E Chaudhry F McBride R : SARS-CoV-2 viral load predicts COVID-19 mortality. *Lancet Respir Med.* 2020;8(9): e70. 10.1016/S2213-2600(20)30354-4 32771081 PMC7836878

[40] Blanco-Melo D Nilsson-Payant BE Liu WC : Imbalanced host response to SARS-CoV-2 drives development of COVID-19. *Cell.* 2020;181(5):1036–1045. e9. 10.1016/j.cell.2020.04.026 32416070 PMC7227586

[41] Watson J Richter A Deeks J : Testing for SARS-CoV-2 antibodies. *BMJ.* 2020;370: m3325. 10.1136/bmj.m3325 32900692

[42] Zuo J Dowell AC Pearce H : Robust SARS-CoV-2-specific T cell immunity is maintained at 6 months following primary infection. *Nat Immunol.* 2021;22(5):620–626. 10.1038/s41590-021-00902-8 33674800 PMC7610739

[43] Grifoni A Weiskopf D Ramirez SI : Targets of T cell responses to SARS-CoV-2 coronavirus in humans with COVID-19 disease and unexposed individuals. *Cell.* 2020;181(7):1489–1501. e15. 10.1016/j.cell.2020.05.015 32473127 PMC7237901

[44] Gaebler C Wang Z Lorenzi JCC : Evolution of antibody immunity to SARS-CoV-2. *Nature.* 2021;591(7851):639–644. 10.1038/s41586-021-03207-w 33461210 PMC8221082

[45] Menni C Valdes AM Freidin MB : Real-time tracking of self-reported symptoms to predict potential COVID-19. *Nat Med.* 2020;26(7):1037–1040. 10.1038/s41591-020-0916-2 32393804 PMC7751267

[46] Castillo-Olivares J Wells DA Ferrari M : Analysis of serological biomarkers of SARS-CoV-2 infection in convalescent samples from severe, moderate and mild COVID-19 cases. *Front Immunol.* 2021;12: 748291. 10.3389/fimmu.2021.748291 34867975 PMC8640495

[47] Butler SE Crowley AR Natarajan H : Distinct features and functions of systemic and mucosal humoral immunity among SARS-CoV-2 convalescent individuals. *Front Immunol.* 2021;11: 618685. 10.3389/fimmu.2020.618685 33584712 PMC7876222

[48] Nelde A Bilich T Heitmann JS : SARS-CoV-2-derived peptides define heterologous and COVID-19-induced T cell recognition. *Nat Immunol.* 2021;22(1):74–85. 10.1038/s41590-020-00808-x 32999467

[49] da Silva Antunes R Pallikkuth S Williams E : Differential T-cell reactivity to endemic Coronaviruses and SARS-CoV-2 in community and Health Care Workers. *J Infect Dis.* 2021;224(1):70–80. 10.1093/infdis/jiab176 33822097 PMC8083569

[50] Griffith GJ Morris TT Tudball MJ : Collider bias undermines our understanding of COVID-19 disease risk and severity. *Nat Commun.* 2020;11(1): 5749. 10.1038/s41467-020-19478-2 33184277 PMC7665028

[51] Nattrass RG Krafft L Zjablovskaja P : The effect of age on the magnitude and longevity of Th1-directed CD4 T-cell responses to SARS-CoV-2. *Immunology.* 2022;166(3):327–340. 10.1111/imm.13475 35396852 PMC9111694

[52] Cohen CA Li APY Hachim A : SARS-CoV-2 specific T cell responses are lower in children and increase with age and time after infection. *Nat Commun.* 2021;12(1): 4678. 10.1038/s41467-021-24938-4 34326343 PMC8322064

[53] Belchior-Bezerra M Lima RS Medeiros NI : COVID-19, obesity, and immune response 2 years after the pandemic: a timeline of scientific advances. *Obes Rev.* 2022;23(10): e13496. 10.1111/obr.13496 35837843 PMC9349458

[54] Zulu MZ Sureshchandra S Pinski AN : Obesity correlates with pronounced aberrant innate immune responses in hospitalized aged COVID-19 patients. *Front Immunol.* 2021;12: 760288. 10.3389/fimmu.2021.760288 34707619 PMC8542887

[55] Soffer S Glicksberg BS Zimlichman E : The association between obesity and peak antibody titer response in COVID-19 infection. *Obesity (Silver Spring).* 2021;29(9):1547–1553. 10.1002/oby.23208 33945220 PMC8242567

[56] Frasca D Reidy L Romero M : The majority of SARS-CoV-2-specific antibodies in COVID-19 patients with obesity are autoimmune and not neutralizing. *Int J Obes (Lond).* 2022;46(2):427–432. 10.1038/s41366-021-01016-9 34744161 PMC8572364

[57] Harris PA Taylor R Thielke R : Research Electronic Data Capture (REDCap)--a metadata-driven methodology and workflow process for providing translational research informatics support. *J Biomed Inform.* 2009;42(2):377–381. 10.1016/j.jbi.2008.08.010 18929686 PMC2700030

