# Supplementary material for: SARS-CoV-2 memory response in non-hospitalised cases: immunology in the context of a population-based cohort study
Source: Wellcome Open Res. 2026 Jun 13;9:580. Originally published 2024 Oct 11. [Version 2] doi: 10.12688/wellcomeopenres.22942.2 (PMC13550896; doi:10.12688/wellcomeopenres.22942.2)
Supplement: Peer review history

# Reviewer report: Reviewer response for version 2

**DOI:** 10.21956/wellcomeopenres.29671.r158113

**Published:** 2026-07-07

**Authors:** Sadhana Sharma

**Affiliations:** 1 All India Institute of Medical Sciences, Patna, India

The authors have satisfactorily addressed my previous comments. The manuscript is well written, the methodology is rigorous, and the conclusions are appropriately supported by the data. The study makes a valuable contribution by characterizing both humoral and cellular immune memory following mild SARS-CoV-2 infection within a well-established longitudinal population-based cohort.

 I have no further major concerns and recommend that the article be **approved** for indexing in its current form.

Is the work clearly and accurately presented and does it cite the current literature?

Partly

If applicable, is the statistical analysis and its interpretation appropriate?

I cannot comment. A qualified statistician is required.

Are all the source data underlying the results available to ensure full reproducibility?

Partly

Is the study design appropriate and is the work technically sound?

Yes

Are the conclusions drawn adequately supported by the results?

Yes

Are sufficient details of methods and analysis provided to allow replication by others?

No

Reviewer Expertise:

Biochemistry and Molecular Biology

I confirm that I have read this submission and believe that I have an appropriate level of expertise to confirm that it is of an acceptable scientific standard.

# Reviewer report: Reviewer response for version 1

**DOI:** 10.21956/wellcomeopenres.25262.r114788

**Published:** 2024-12-30

**Authors:** Angela Crawley

**Affiliations:** 1 University of Ottawa, Ottawa, Ontario, Canada

This was a very interesting cohort opportunity to study pre- and pan-pandemic responses. The value of such longitudinal efforts is critically important to informing appropriate and effect public health responses to threats to human health. 

 The use of multi-parameter ELISAs and the neutralization assay further strengthens the data value.

 Statistical analysis of immune response correlations are very simple, and not integrative, allowing only for univariate interpretations. Although, Fig 8 does go into some detail on this point. An understanding of how multiple variables work together to drive particular immune responses would be achieved through more in-depth integrative, systems analyses and perhaps other such studies could be referenced (e.g., Cuperlovic-Culf M. et al., iScience 2024).

 Color schemes used across figures are not consistent, or not clarified (e.g., Fig 4 - what is red and black?)

Is the work clearly and accurately presented and does it cite the current literature?

Yes

If applicable, is the statistical analysis and its interpretation appropriate?

I cannot comment. A qualified statistician is required.

Are all the source data underlying the results available to ensure full reproducibility?

Yes

Is the study design appropriate and is the work technically sound?

Yes

Are the conclusions drawn adequately supported by the results?

Yes

Are sufficient details of methods and analysis provided to allow replication by others?

Yes

Reviewer Expertise:

immunology, biobanking, liver disease

I confirm that I have read this submission and believe that I have an appropriate level of expertise to confirm that it is of an acceptable scientific standard.

## References

[1] : Multivariate analyses and machine learning link sex and age with antibody responses to SARS-CoV-2 and vaccination. 10.1016/j.isci.2024.110484 10.1016/j.isci.2024.110484 PMC1132802039156648

# Response

**Published:** 2026-06-01

**Authors:** Nic Timpson

**Affiliations:** University of Bristol, UK

Please find below a response to review comments (responses flagged as “**”):  

**Comments to Authors:**

*This was a very interesting cohort opportunity to study pre- and pan-pandemic responses. The value of such longitudinal efforts is critically important to informing appropriate and effect public health responses to threats to human health.*

*The use of multi-parameter ELISAs and the neutralization assay further strengthens the data value.*

**We were delighted to receive this feedback and also overall approval for the manuscript as submitted. The overarching aim of this work is to use existing data and illustrative examples to demonstrate the utility of longitudinal data in the context of charting immunological response to a novel infection challenge. This clearly sets out the scope of the paper and hence the response to our second review. Thanks again for the comments.

**Reviewer comments:**

*"Statistical analysis of immune response correlations are very simple, and not integrative, allowing only for univariate interpretations. Although, Fig 8 does go into some detail on this point. An understanding of how multiple variables work together to drive particular immune responses would be achieved through more in-depth integrative, systems analyses and perhaps other such studies could be referenced (e.g., Cuperlovic-Culf M. et al., iScience 2024)."*

**As mentioned above, the purpose – hence scope – of this manuscript was to present the potential for the analysis of life course factors and potential contributions to the immunological responses to novel challenge. The analysis brought together are for this purpose and whilst we completely agree with the appeal of using these data for more integrative analyses, that is out of scope for this specific report. We understand the potential for the types of analyses referred to and we agree that it would be interesting to evaluate in future work, where sample size allows, how multiple variables interact in relation to immune responses. Here we consider immune response to infection in a relatively small immune-naïve, non-hospitalised cohort.

**Further work using these data and considering a more comprehensive description of longitudinal immunological response has been published alongside this work (albeit with less incorporation of life course data). This can be seen here:  Baum HE et al. Hybrid B- and T-Cell Immunity Associates With Protection Against Breakthrough Infection After Severe Acute Respiratory Syndrome Coronavirus 2 Vaccination in Avon Longitudinal Study of Parents and Children (ALSPAC) Participants. J Infect Dis. 2025 Aug 14;232(2):e327-e340. doi: 10.1093/infdis/jiaf246. Erratum in: J Infect Dis. 2026 Mar 18:jiag161. doi: 10.1093/infdis/jiag161. PMID: 40392230; PMCID: PMC12349941.**

*"Color schemes used across figures are not consistent, or not clarified (e.g., Fig 4 - what is red and black?)  "*

**Thanks for this comment. Overall, the colour schemes are not the same (or linked) across the various figures because they are not attempting to suggest that there are common threads running between each presentation. We wanted figures to be essentially stand-alone and to this extent would suggest that the colour schemes remain as they are.  We do, however, concede that the labelling of figure 4 is not clear. To this end, we have incorporated a new legend into this figure and submitted it as part of this response.

# Reviewer report: Reviewer response for version 1

**DOI:** 10.21956/wellcomeopenres.25262.r114015

**Published:** 2024-12-20

**Authors:** Sadhana Sharma

**Affiliations:** 1 All India Institute of Medical Sciences, Patna, India

**Comments to Authors:**

 The study entitled “ SARS-CoV-2 memory response in non-hospitalised cases:immunology in the context of a population-based cohort Study” provides valuable insights into the immune response following natural SARS-CoV-2 infection, particularly in mild and asymptomatic cases, an area that has been under explored. The use of the ALSPAC cohort is a significant strength, enabling the analysis of long-term health data and the exploration of prior health factors that could influence immune responses. The study's comprehensive approach measuring antibodies, neutralizing antibodies, and T-cell responses adds depth to our understanding of immune memory. Additionally, the use of both self-reported symptoms and a validated algorithm to classify symptomatic and asymptomatic cases strengthens the analysis of symptom severity and its correlation with immune responses. However, there are several areas that need further clarification and refinement to ensure the study's conclusions are robust and reliable.

1. First, while the paper acknowledges potential biases, such as index-event and collider biases, a more detailed discussion of how these biases could influence the results is needed. Specifically, the mention of eczema as a potential protective factor for COVID-19 requires further explanation, given the study's design limitations.
2. Additionally, the reliance on self-reported symptom data presents a limitation, as it could result in underreporting or misreporting. This may affect the interpretation of the association between symptom severity and immune response. The authors should address these limitations more thoroughly and consider incorporating more objective markers, such as clinical assessments or biomarkers, in future studies.
3. The paper also notes that viral load and the innate immune response were not measured, yet these factors could be important confounders. The absence of these data should be discussed in more detail, and the authors should explain how the lack of this information might influence the interpretation of the findings.
4. Furthermore, while the study investigates the impact of BMI, it does not explore other potential modifiers of immune response, such as sex, comorbidities, or socio-economic factors. Expanding the analysis to include these variables could provide a more comprehensive understanding of factors that influence immune responses to SARS-CoV-2.

**Suggestions for Improvement:**

1. Provide more detail on how biases, particularly index-event and collider biases, may affect the results and steps taken to mitigate them.
2. Discuss the limitations of self-reported symptom data in more detail and consider using more objective markers in future studies.
3. Elaborate on the absence of viral load and innate immune response data and how this could impact the results.
4. Expand the analysis to include other health factors, such as comorbidities, age, and socio-economic status.
5. The study makes a significant contribution by exploring immune memory in non-hospitalized cases, a less-studied area.
6. Minor revisions focusing on simplifying technical jargon and expanding on the implications of findings could further enhance the article’s impact.

Is the work clearly and accurately presented and does it cite the current literature?

Partly

If applicable, is the statistical analysis and its interpretation appropriate?

I cannot comment. A qualified statistician is required.

Are all the source data underlying the results available to ensure full reproducibility?

Partly

Is the study design appropriate and is the work technically sound?

Yes

Are the conclusions drawn adequately supported by the results?

Yes

Are sufficient details of methods and analysis provided to allow replication by others?

No

Reviewer Expertise:

Immunology, Molecular Biology, Biochemistry and Proteomics

I confirm that I have read this submission and believe that I have an appropriate level of expertise to confirm that it is of an acceptable scientific standard, however I have significant reservations, as outlined above.

# Response

**Published:** 2026-06-01

**Authors:** Nic Timpson

**Affiliations:** University of Bristol, UK

Please find below a response to review comments (responses flagged as “**”):  

**Comments to Authors:**

*“The study entitled “ SARS-CoV-2 memory response in non-hospitalised cases:immunology in the context of a population-based cohort Study” provides valuable insights into the immune response following natural SARS-CoV-2 infection, particularly in mild and asymptomatic cases, an area that has been under explored. The use of the ALSPAC cohort is a significant strength, enabling the analysis of long-term health data and the exploration of prior health factors that could influence immune responses. The study's comprehensive approach measuring antibodies, neutralizing antibodies, and T-cell responses adds depth to our understanding of immune memory. Additionally, the use of both self-reported symptoms and a validated algorithm to classify symptomatic and asymptomatic cases strengthens the analysis of symptom severity and its correlation with immune responses.”*

**We acknowledge comments flagging the potential for type of immune system study – nested within a longitudinal population study – thank you.**

**Reviewer comments**

*“First, while the paper acknowledges potential biases, such as index-event and collider biases, a more detailed discussion of how these biases could influence the results is needed. Specifically, the mention of eczema as a potential protective factor for COVID-19 requires further explanation, given the study's design limitations.”*

** We thank the reviewer for this comment and – as in the manuscript – acknowledge the presence of possible limitations to the data and interpretation of results as a result of a series of potential limitations. It is possible to go through these and to speculate as to the potential possible effects they may have had on results presented. However, for many of these potential limitations, it is not possible to measure/assess these complications empirically.

**Taking an example; the presence of collider bias induced by the structure of the sample available for analysis may have contributed to the estimates presented - whilst it is important to state the potential of this as a limiting phenomenon, it is almost impossible to quantify or avoid this type of effect with the bounds of the existing sample and scope of the paper.**

**Our approach to this problem is two-fold. Firstly - and from an analytical point of view - one of the benefits of deploying this type of serological assessment within a longitudinal study such as ALSPAC is the availability of additional – potentially biologically related – covariables able to allow us to examine patterns of association across a wider phenotype collection and where real and potentially artefactual associations can be compared to main trends (through time or across age/generation). To this end, we included – amongst other measures – the assessment of immune system related phenotypes such as asthma, allergy and atopic disease - each which carry some priors as to associations with immune response. In the G1 participants, eczema (140 months), hay fever (age 11), allergic sensitivity via a skin prick test (age 7) were examined and there were suggestive relationships between childhood eczema and a lower memory response (anti-N antibodies for those who had eczema at 140 months). These point estimates were compelling, though were relatively low in power and there is certainly no direct causal inference that can be drawn from these results. It is tempting to consider that early immunological exposures and associated biological events may have an effect on later immune responsiveness, which remains for at least some part of life, however we were keen not to over interpret any associations. Secondly, we provide an open and clear analysis of well described data and also to provide access to those data in order that they can be analysed again and potentially alongside other data sets with differing sampling frame structure and limitations (hence informative to overall inference). This does not deny the presence of possible sample complications, rather uses available data openly and transparently to add to the overall evidence base within the scope of the oiginally designed work.**

**Overall, given the nature of observational results, the variable nature of relationships across ages and generations and the need for further examination of this type of result within large population studies with detailed measures and samples across periods of frequent infection, we still feel that it is most appropriate not to expand on speculative explanations as to why these relationships are present, but to present simple analyses/descriptions of this specific project in a context where resources are available for further examination.**

*“Additionally, the reliance on self-reported symptom data presents a limitation, as it could result in underreporting or misreporting. This may affect the interpretation of the association between symptom severity and immune response. The authors should address these limitations more thoroughly and consider incorporating more objective markers, such as clinical assessments or biomarkers, in future studies.”*

** This study looks at non-hospitalised COVID-19 cases, whose infection occurred before December 2020 and was identified via retrospective serological testing. For many of the cases, the date of infection was unknown as infections were mild or asymptomatic and testing was unavailable or limited during the early stages of the pandemic. To mitigate against the limitations of only being able to collect self-reported symptom data for this period, we used three different measures of symptom severity: the first assigning a case as asymptomatic or symptomatic based on the Menni algorithm; the second based on reported loss of taste or smell; and the third based on the number of symptoms recorded in the first month of a maximum jump in number of symptoms.

**Indeed, in future studies, if LFD testing is available from the outset, participants can be asked to test regularly and attend a clinic at the time of infection to more accurately assess symptoms with a clinician or to collect biomarker samples.**

*“The paper also notes that viral load and the innate immune response were not measured, yet these factors could be important confounders. The absence of these data should be discussed in more detail, and the authors should explain how the lack of this information might influence the interpretation of the findings.”*

** We agree that viral load, innate virus sensing (including type-I IFN signalling) and cellular innate immunity (e.g. neutrophils, macrophages, NK-cells) are important players in SARS-CoV-2 infection. The role of innate immunity is twofold: it provides a first line of defence against SARS-CoV-2 infection by recognising and restricting virus replication and it primes adaptive T and B cell responses which in turn generate an antigen-specific effector response and long-lived memory T and B cells. Characterisation of all these parameters would require a different study design where participants are recruited at the time of infection and viral load, virus sensing mechanisms and cellular innate immunity are comprehensively measured. These aspect therefore remain out of scope for this investigation and we are keen not to over complicate the presentation of the study or the analyses undertaken, though very happy to acknowledge this characteristic of the study here in review and as in the paper currently.

*“Furthermore, while the study investigates the impact of BMI, it does not explore other potential modifiers of immune response, such as sex, comorbidities, or socio-economic factors. Expanding the analysis to include these variables could provide a more comprehensive understanding of factors that influence immune responses to SARS-CoV-2.”*

** The immune variable analyses were broken down by sex, please see Supplementary Figures 1-3 and the discussion in the Results section. Age was evaluated throughout by considering separately the G0 cohort of parents (mean age 58.5 years) and G1 cohort of offspring (mean age 28.3 years). The ALSPAC cohort is a generally healthy cohort and we did not have data or sufficient sample size to test associations with common comorbidities such as diabetes, cancer and hypertension. In this study, we did not look at socio-economic status and considered this and further covariables to be outside the scope of this work specifically. ALSPAC data on index of multiple deprivation (IMD) is only available for a subset of participants, namely the mothers in generation G0.
